# From Apo to Ligand-Bound: Unraveling PPARγ-LBD Conformational Shifts via Advanced Molecular Dynamics

**DOI:** 10.1021/acsomega.4c11128

**Published:** 2025-02-17

**Authors:** Emanuele Falbo, Pietro Delre, Antonio Lavecchia

**Affiliations:** Department of Pharmacy, “Drug Discovery Laboratory”, University of Naples Federico II, via Domenico Montesano 49, I-80131 Naples, Italy

## Abstract

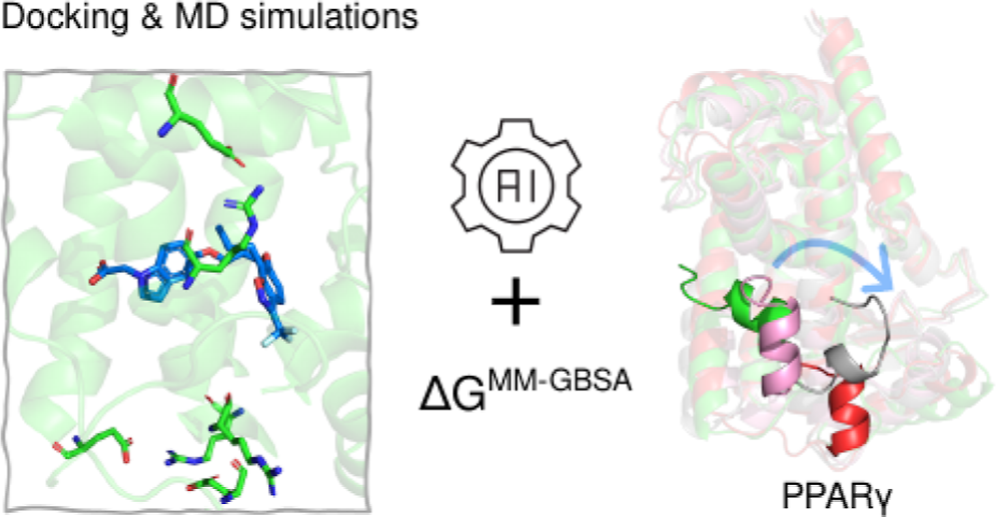

Peroxisome proliferator-activated receptor gamma (PPARγ) is a nuclear receptor whose ligand-induced conformational changes, primarily driven by helix 12 (H12) repositioning, regulate transcriptional activity. However, the precise mechanism remains elusive. In this study, we performed classical molecular dynamics (cMD) simulations of the PPARγ ligand binding domain (LBD) in complex with two agonists (BRL, 3EA), a partial agonist (GW0072), and an antagonist (EKP), generating 3 μs trajectories for each system. To gain deeper insights, we integrated machine learning-assisted clustering with MD simulations, revealing a favorable trend in binding free energy (Δ*G*_b_), suggesting enhanced complex stability. A case study on EKP demonstrated that, despite fitting within the binding site, it failed to induce rapid LBD or H12 rearrangements in the apo agonist-induced conformation. Additionally, we investigated the apo-state conformations of PPARγ-LBD influenced by agonist and antagonist ligands, utilizing cMD and Gaussian accelerated molecular dynamics (GaMD) over a cumulative 6 μs (3 μs cMD + 3 μs GaMD). Key residues known to modulate PPARγ function upon mutation were analyzed, and simulations confirmed the high stability of both apo and ligand-bound conformations. Notably, in the apo state, specific H12 residues interacted with other PPARγ-LBD regions, preventing disorder and abrupt transitions. These findings guided the selection of collective variables (CVs) for well-tempered metadynamics (WT-MetaD) simulations, which-in the apo-agonist state-captured the H12 shift from agonist- to antagonist-like conformations, consistent with resolved X-ray structures. Overall, this computational framework provides novel insights into PPARγ-LBD conformational dynamics and establishes a valuable approach for rationally assessing the effects of modulators on PPARγ activity.

## 1 Introduction

### 1.1 Background

**Figure 1:**
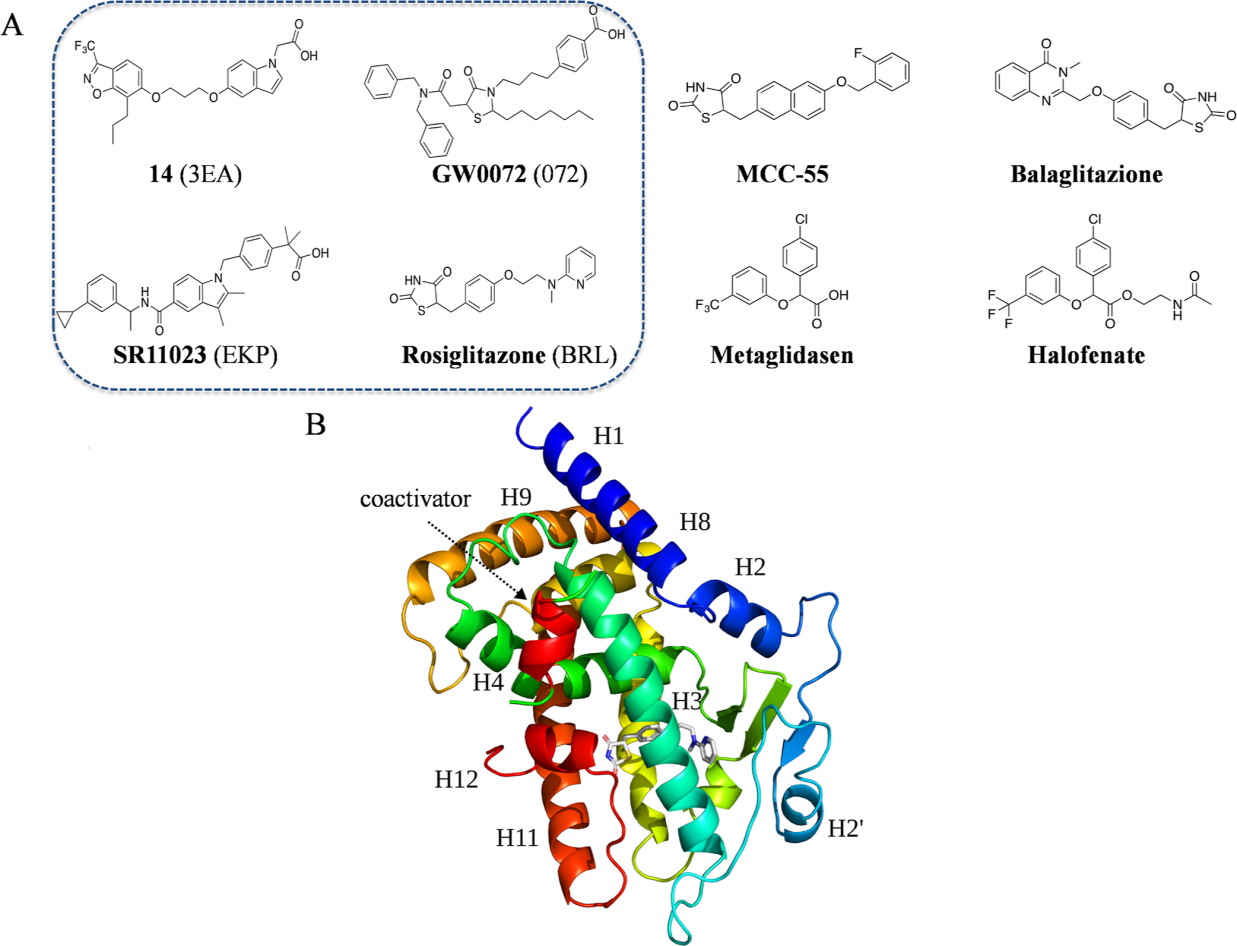
(A) 2D structures of representative compounds targeting PPARγ. The selected ligands for simulations are highlighted in the blue circle: agonists (BRL and 3EA) with and without coactivator, a partial agonist (GW0072), and an antagonist (EKP). (B) Overall structure of the PPARγ ligand-binding domain (LBD) with the BRL ligand (shown in gray licorice) bound to the orthosteric site, alongside the coactivator protein (colored red). Key α-helices are labeled for clarity.

PPARγ primarily functions through obligate heterodimerization with retinoid X receptor alpha (RXRα). This heterodimeric complex binds to specific DNA sequences called peroxisome proliferator response elements (PPREs) in regulatory regions of hundreds of genes. This binding promotes transcriptional activation, leading to the expression of proteins that produce physiological effects. Pharmaceuticals targeting PPARγ have generated billions of dollars.^1,2^ Thiazolidinediones (TZD) like Pioglitazone and Rosiglitazone (Figure 1A) were the first designed ligands, acting as full synthetic receptor agonists commercialized around 1999–2000, often in combination with metformin for antidiabetic effects. However, their initial success was tempered by side effects such as weight gain, fluid retention, and increased risk of heart failure, limiting their clinical use.^3^ To address these issues, researchers are developing second-generation PPARγ ligands with partial agonist or antagonist properties,^4−8^ including MCC-555, DRF-2593 (balaglitazone), metaglidasen, and halofenate (Figure 1A), some of which are in Phase II and III clinical trials.^9−11^ PPARγ ligands not only benefit diabetes treatment but also show promise in other areas. Evidence suggests that PPARγ overexpression can accelerate cancer progression, leading to interest in selective PPARγ antagonists for cancer therapy.^12^ Additionally, its involvement in regulating neuroinflammation suggests potential applications in neurodegenerative diseases such as amyotrophic lateral sclerosis (ALS) and Parkinson’s disease.^12^ Ongoing research into PPARγ modulation underscores the importance of understanding ligand–receptor interactions to develop safer and more effective drugs.^13^

### 1.2 Overall Structure and Ligand Binding Domain Motion

PPARγ consists of a five-domain structure that functions as a transcription factor, regulating gene expression in response to specific ligands. The N-terminal regulatory domain modulates transcriptional activity, while the DNA binding domain recognizes PPREs. The hinge domain, poorly conserved, serves as a flexible connection between the DNA and the LBD. The LBD-PPARγ is a pivotal focus in pharmaceutical research due to its role in ligand binding and regulatory mechanisms. A critical site within the LBD is the activation function 2 (AF-2), which includes H12, H3, H4 and H5 (Figure 1B). AF-2 facilitates interaction and recruitment of coactivator molecules, thereby influencing gene expression.^14^ Compared to other nuclear receptors, PPARγ-LBD features a larger pocket that accommodates a broader array of ligands, including endogenous lipids and fatty acids.^14^ Upon agonist binding in the LBD, stabilization of H12 within the AF-2 region occurs through direct hydrogen bond (H-bond) interactions, facilitating coactivator recruitment.^14−16^ Studies have shown that in its apo form, the LBD, particularly H12, exhibits high mobility. This dynamic behavior has been extensively investigated using chemical cross-linking mass spectrometry (MS) and nuclear magnetic resonance (NMR), revealing various conformations, including an antagonist-bound state.^17^ Recent findings suggest that conformational selection plays a role in the ligand-binding mechanism of modulators, stabilizing a specific conformation from a dynamic ensemble of active and inactive/repressive states.^18−20^ Additionally, a recent theory, utilizing protein NMR chemical shift perturbation (CSP) analysis, proposes that agonist binding to the PPARγ-LBD occurs via an induced fit mechanism, comprising an initial fast kinetic step followed by a slower conformational change.^21^

### 1.3 Aims of the Study

**Figure 2:**
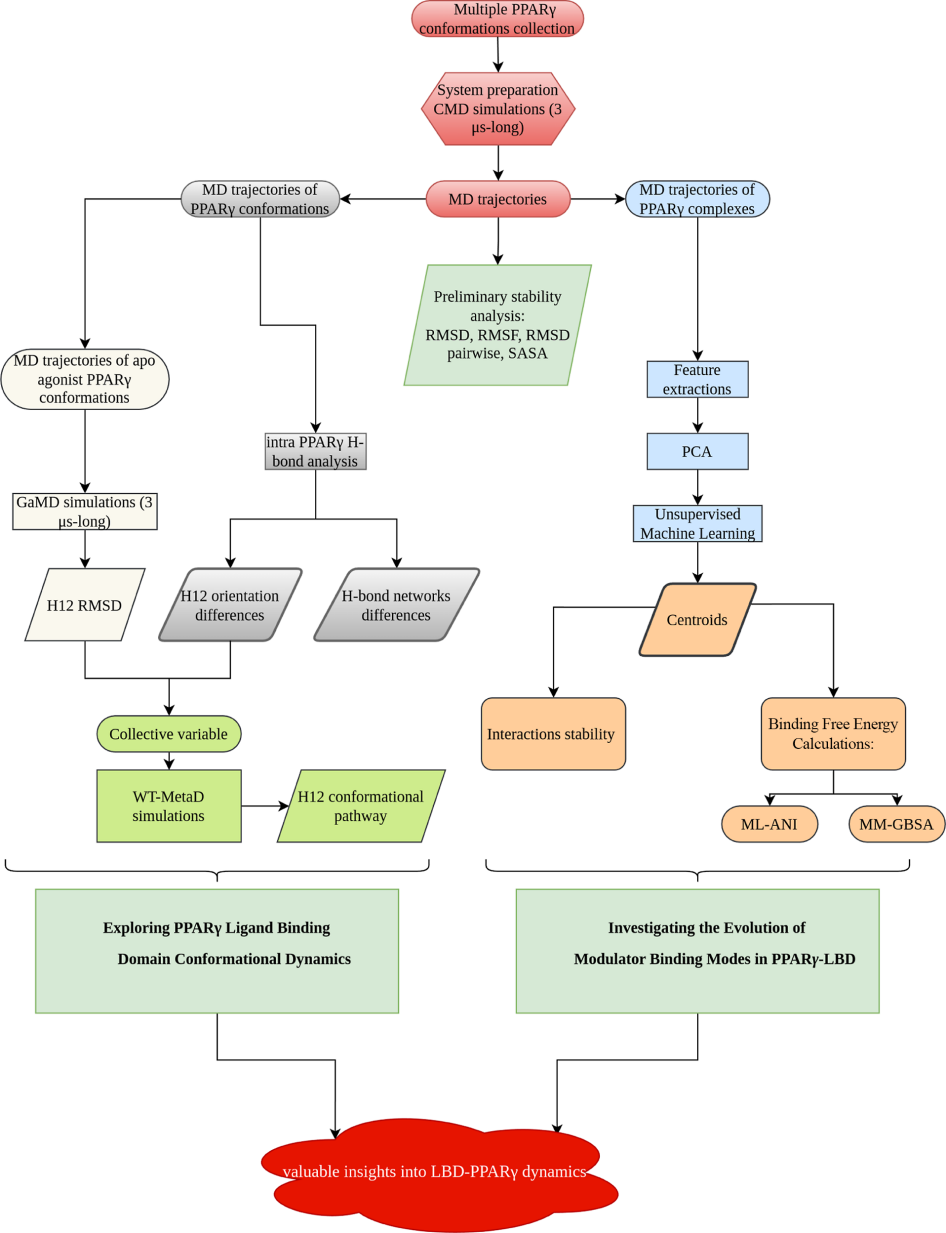
Figure illustrating the workflow diagram including the computational methodologies used in this study to investigate the dynamics of the PPARγ ligand binding domain (LBD) and the evolution of the modulator binding modes.

We present a protocol integrating cMD and WT-MetaD simulations with ML algorithms^22^ to investigate the binding mechanism and conformational changes of PPARγ-LBD (Figure 2). The simulations include various conformations, such as ligand-bound PPARγ crystal structures: two agonist-bound complexes (PDB: 2ATH(23) and PDB: 3DZY(24)), one antagonist-bound complex, and one partial agonist-bound complex (PDB: 4PRG(25)). Notably, the only solved crystal structure of antagonist-induced PPARγ, 6C5T,^26^ has not been explored through MD before. Employing this workflow, we conducted molecular docking studies of the antagonist SR11023 (referred to as EKP) in the agonist-bound conformation (PDB: 3DZY) to gain insights into the ligand-induced conformational changes of the binding site. While cMD simulations revealed only minor structural rearrangements, ML-refined binding free energy calculations effectively captured ligand accommodation within the binding pocket. This was particularly evident when comparing free energy values derived from clustered conformations to those obtained from the crystal structures. Furthermore, our workflow demonstrated that EKP preferentially stabilizes the antagonist-induced conformation over the agonist-bound state, offering a promising foundation for further experimental validation. This approach serves as a valuable tool for rationally investigating the influence of PPARγ modulators on its conformational dynamics.

Additionally, we performed cMD simulations on the apo forms by removing the modulators from the binding pocket to evaluate the stability of both agonist- and antagonist-induced conformations. A detailed analysis of MD trajectories identified 14 shared interactions between all systems, with a significant difference in the H-bonds formed by six key residues (R288, E295, R280, E276, R357, and E460) depending on the ligand bound. While mutations in R288 and R357 have been linked to impaired PPARγ function associated with metabolic disorders and cancer,^27^ our study offers the first comprehensive characterization of their cooperative roles alongside E295, R280, E276, and E460 in regulating PPARγ-LBD dynamics. Both cMD and GaMD simulations revealed significant stability of H12, even in the apo forms, maintaining the ligand-induced conformation without drastic helix shifts. Finally, WT-MetaD simulations offered valuable insights into H12 conformational transitions, highlighting the differences in movement between apo and bound states and reinforcing the hypothesis of a mixed-induced adaptation mechanism.^21^

## 2 Methods

### 2.1 cMD Simulations

MD simulations were performed on the following X-ray downloaded from the Protein Data Bank (PDB):

i. human PPARγ in complex with the agonist 3EA (PDB: 2ATH(23)), abbreviated as agonist/3EA.
ii. Human PPARγ in complex with the antagonist EKP (PDB: 6C5T(26)), abbreviated as antagonist/EKP.
iii. Human PPARγ in complex with the partial agonist GW0072 (PDB: 4PRG(25)), abbreviated as partial agonist/GW0072.
iv. Human PPARγ in complex with the agonist BRL (3DZY^24^), abbreviated as agonist/BRL.
v. Apo forms of human PPARγ (antagonist, agonist, and partial-agonist) derived from ligand elimination in the corresponding PDB structures: 6C5T,^26^2ATH,^23^ and 4PRG,^25^ respectively.
vi. A complex generated by docking of EKP into the agonist conformation of BRL (PDB: 3DZY(24)), abbreviated as agonist/EKP.

For homocomplexes and heterocomplexes, only one monomer of PPARγ was extracted. In the case of the 3DZY complex, we isolated the PPARγ-LBD and the NCOA2 peptide fragment. Missing parts of proteins were reconstructed using Prime,^28^ and all structures underwent preliminary preparation with the Protein Preparation Wizard (PPW).^29^ Specifically, PPW added missing hydrogen atoms, reconstructed incomplete side chains, assigned the ionization states at physiological pH, set the orientation of any misoriented groups (N, Q, and H residues), optimized the H-bond network, and performed a restrained minimization using PPW default settings. Holo and apo forms of human PPARγ were prepared employing explicit solvent (TIP3P water model) with periodic boundary conditions. The ff14SB AMBER force field^30^ was used for proteins. For ligands modeling, there are different traditional^30−32^ or novel^33−35^ force field approaches, in this work, the ligands were processed using the general AMBER force field (GAFF2).^36^ All classical simulations were performed with AMBER 2022 software.^37,38^ Initially, the systems were equilibrated by restraining the protein to its initial position with harmonic restraints (20 kcal/mol). Each system underwent minimization, followed by heating to 298 K for 250 ps in a constant-volume ensemble (*NVT*). The numerical integration step was set to 2.0 fs, and the Langevin thermostat was used with a 5.0 ps^–1^ collision frequency. The systems were then allowed to relax for an additional 500 ps using a constant-pressure ensemble (*NPT*) at 298 K and 1 atm. In all cases, the bond lengths involving hydrogen atoms were kept at their equilibrium distances using the SHAKE algorithm.^39^ Distance cutoffs were applied at 10.0 Å to compute the van der Waals interactions. Long-range electrostatics were computed with the particle mesh Ewald method. Constraints on the solute atoms were removed, and another trajectory was generated with constant density under the same conditions for a duration of 5 ns. Subsequently, production runs were conducted in the *NVT* ensemble at 298 K for 3 μs. The coordinates of the production trajectories were saved every 0.1 ns for further analysis. All trajectories were analyzed using in-house codes using the MDAnalysis library to calculate H-bond occupancies, root-mean-square deviation (RMSD) and root-mean-square fluctuation (RMSF) values.^40,41^

### 2.2 GaMD

We extended the cMD simulations of the apo forms using GaMD.^42,43^ This technique boosts the total potential following a Gaussian distribution, allowing accurate reweighting using the cumulative expansion to the second order. GaMD increases sampling of conformational space without requiring predefined reaction coordinates. We applied a dual boost on the dihedral and total potential energies (*V*) by setting the threshold energy at the lower bound *E* = *V*_max_. Potential energies were collected from 200,000 conventional molecular dynamics preparation steps and 1,000,000 initial conventional molecular dynamics simulation steps to calculate the maximum, minimum, mean, and standard deviation of *V* (*V*_max_, *V*_min_, *V*_avg_, and σ*V*). These quantities were then used to perturb the potential by running the default GaMD algorithm implemented in the AMBER software. The upper limit of the standard deviation of the boost potential, which allows for accurate reweighting, was left at the default of 6.0 kcal/mol. All cMD settings remained unchanged. Production runs were conducted in the *NVT* ensemble at 298 K for 3 μs, with trajectory saved every 0.1 ns for further analysis.

### 2.3 Docking Experiments

The EKP ligand was processed using LigPrep^44^ to generate all tautomers and ionization states at a pH of 7.0 ± 2.0. The agonist-induced conformation (3DZY^24^) was used for docking simulations using the Schrödinger and the GLIDE^45^ v.6.5 algorithm. A cubic grid having an edge of 30 Å for the outer box and 10 Å for the inner box (GLIDE) was built centered on the BRL ligand. The receptor protein was kept fixed, while the ligand was allowed complete conformational flexibility during simulations. The OPLS_2005_ force field and the standard precision (SP)^45^ protocol with default settings were employed.

### 2.4 Metadynamics Simulations

The apo agonist conformation extracted from PDB: 2ATH,^23^ along with agonist/3EA, partial agonist/GW0072 and antagonist/EKP complexes were subjected to WT-MetaD simulations using Desmond 4.4 software on NVIDIA T1000 8GB graphic cards (GPUs). A set of Collective Variables (CVs) was employed to capture translational, rotational, and conformational motions of the ligand within the PPARγ-LBD. Gaussian potentials were added every 2 ps with a constant height of 0.4 kcal/mol. The width of these Gaussians was set to one-third of the average fluctuations of the CV during a free MD run, approximately 0.20 Å. A restraining wall was positioned at the CV value corresponding to the sum of the largest dimension of the complex plus either 30 or 40 Å, to prevent the CV from deviating excessively. Although metadynamics simulations involve nonequilibrium physical processes, statistical errors, arising from the random nature of the simulations, can be minimized by tracking the change in the CVs, barrier height, and free energy surface (FES) across multiple independent runs.^46^

Previous studies,^47^ have indicated that converging the full free energy landscape is feasible but can be very time-consuming and may require system-specific selection of CVs. In this study, we performed several independent WT-MetaD simulations to calculate the average FES. Each system was replicated into multiple replicas which differ from each other only by the random seed used in the input configuration file. Each replica underwent multiple stages as instructed in the msj file: (i) Brownian dynamics in the *NVT* ensemble at 10 K, small timesteps, restraints on solute heavy atoms and 100 ps. (ii) Berendsen dynamics of *NVT* ensemble at 10 K, with small timesteps and restraints on solute heavy atoms and 12 ps. (iii) Berendsen dynamics in the *NPT* ensemble at 10 K and restraints only on solute heavy atoms and 12 ps. (iv) Berendsen dynamics of *NPT* ensemble at 310 K and restraints on solute heavy atoms and 12 ps. (v) Berendsen dynamics of *NPT* ensemble at 310 K without restraints and 500 ps. (vi) A production run, consisting of Martyna–Tobias–Klein dynamics of *NPT* ensemble with WT-MetaD that is performed at a temperature of 310 K without restraints for up to 10 or 20 ns to adequately sample the ligand dynamics within the binding site. The final FES was computed by averaging the FES from each replica.

### 2.5 Clustering and Trajectory Analysis

To preserve the meaningful information extracted from MD simulations, we used a previously developed approach,^48^ that utilizes unsupervised learning methods, focusing on appropriate feature spaces. Key features in our study included distances between the ligand and residues within the LBD, as well as their RMSD values, forming our feature matrix. The reference structure for computing RMSD was defined as the initial frame of each simulation. In addition to the specific bond distances between ligand and receptor, we incorporated RMSD values for backbone atoms of the protein, the ligand itself, and the interacting residues within the LBD as geometric features. Specific descriptors for each system are shown in Table S1. To mitigate potential biases due to varying data distributions, each feature was standardized to have a mean of zero and a standard deviation of one.^49−51^ Sampling was conducted at intervals of 10 ps to minimize short-term correlations in clustering.^48,52,53^ Dimensionality reduction was performed using principal component analysis (PCA), retaining components that explained 90% of the total variance. Then, a clustering analysis identified essential components within the feature space, identifying representative frames called cluster centroids. The K-means++ algorithm^54^ was employed throughout this analysis. Its performance was evaluated across multiple runs with values of *k* ranging from 2 to 10, using internal validation criteria such as SilhouetteScore (SI), DunnIndex (DI), Calinski-Harabaszscore (pSF) and Within Sum of Squares error (WSS). SI, DI, and pSF should have a maximum corresponding to the parameter set (the value of *k* in this case) that yields the best clustering, while WSS searches for a change in slope. Through iterative evaluation, the best value of k was determined based on convergence across three out of four criteria, ensuring robust clustering performance. Detailed metrics and results supporting these decisions are provided in the Supporting Information (see Figures S4–S6).

### 2.6 Relative Binding Energy Calculations

Accurately predicting binding free energies remains a challenging task despite decades of research.^55,56^ The Δ*G*_b_ of a molecular system theoretically derives from fundamental thermodynamic principles. However, practical computational estimates require approximations. Techniques like thermodynamic integration (TI) and free-energy perturbations (FEP) typically require numerous sample points along pathways created through Monte Carlo (MC) or MD simulations. However, these simulations could be computationally intensive for large systems. In contrast, end point free energy methods such as the Poisson–Boltzmann surface area (MM-PBSA) and MM-GBSA offer faster alternatives, balancing efficiency with reasonable accuracy.^57−59^ For MM-GBSA rescoring, we selected frames from the full trajectories, docking poses and previously described complexes. These calculations employed predefined dielectric constants, the OPLS4^60^ force field, and the VSGBB^61^ solvation model, with no flexibility allowed to the protein during computations. MM-GBSA was computed using Prime MM-GBSA^62^ according to the following formula
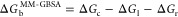
1where Δ*G*_b_^MM-GBSA^ is the energy contribution calculated from the optimized ligand–receptor complex, and Δ*G*_l_ and Δ*G*_r_ are the energy contributions calculated from the optimized free ligand and free receptor, respectively. While MM-GBSA is computationally efficient, its reliance on implicit solvent model and the molecular mechanics (MM) description can limit accuracy. Efforts to enhance MM-GBSA accuracy have explored integrating quantum mechanical descriptions of ligand–protein complexes,^63,64^ and employing a general-purpose neural network-based atomistic potential for organic molecules.^65^ Akkus et al.^66^ have shown that ANI trained with wb97x/6-31G* results can enhance the Δ*G*_b_ by DFT-D3 reproducing with a reasonable confidence experimental data, while maintaining computational efficiency. Therefore, we employed this method, referred to as machine learning-automated network interactions (ML-ANI),^65^ to compute Δ*G*_b_ as follows
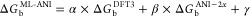
2where α, β, and γ are optimized constants,^66^ and ΔG_b_^DFT3^ and ΔG_b_^ANI–2*x*^ represent the binding energies computed using DFT-D3 (wb97x functional^67^ and 6-31G* basis set^68^) and ANI, respectively. A detailed description of this method can be found in Kocak et al.^66^ More negative values of Δ*G*_b_ indicate a stronger binding affinity. This approach ensures robust validation and enhances our understanding of ligand-binding interactions within the LBD-PPARγ.

## 3 Results and Discussion

To unravel the molecular mechanisms governing the interaction between the LBD-PPARγ receptor and its ligands, we conducted extensive 3 μs long MD simulations on four distinct PPARγ conformations obtained from the PDB: 2ATH(23) (agonist/3EA), 6C5T(26) (antagonist/EKP), 3DYZ (agonist/BRL),^24^ and 4PRG (partial agonist/GW0072).^25^ The resulting trajectories were analyzed using RMSD, RMSF, machine learning clustering, and MM-GBSA rescoring. To further validate our workflow, we performed a case study by docking the antagonist EKP into the agonist/BRL binding site (agonist/BRL-EKP) and applied the same analytical approach.

### 3.1 Investigating the Evolution of Modulator Binding Modes in PPARγ-LBD

#### 3.1.1 Trajectory Analysis of PPARγ-LBD in Complex with Modulators

**Figure 3:**
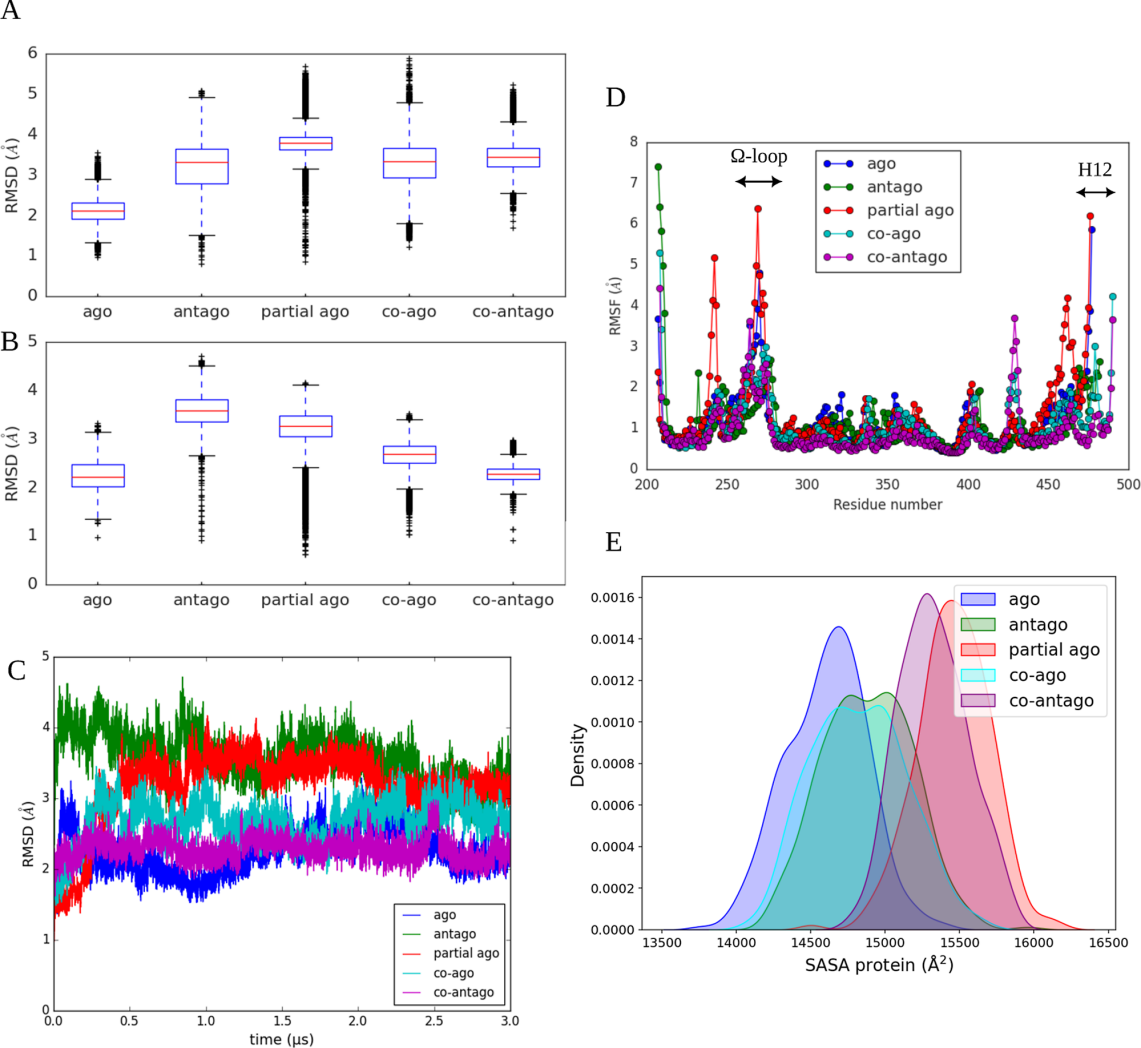
Box plots showing RMSD for (A) ligand and (B) backbone atoms from each MD simulation. Whiskers indicate the range from minimum to maximum values, excluding outliers, with the median represented by a line within the box. (C) RMSD values as a function of time for all simulated complexes. (D) RMSF values for all systems. (E) Density distribution of SASA from the trajectories of selected complexes. Abbreviations: ago = agonist/3EA; partial ago = partial agonist/GW0072; antago = antagonist/EKP; coago = agonist/BRL; coantago = agonist/EKP.

We analyzed the MD trajectories from the simulation of X-ray structures and the case study complex (agonist/EKP). Initially, we computed the time-dependent RMSD of all backbone atoms (Cα, C, O, and N) and ligands aligned to the protein, revealing that both protein and ligand conformations reached equilibrium quickly. The RMSD values for both the ligand and backbone stabilized around their median values. Box plots in Figure 3A,B show a narrow interquartile range (IQR), indicating minimal fluctuations after the initial equilibrium phase. Overall, RMSD analysis confirmed stable conformations and binding poses, with a median RMSD of less than 4 Å across all systems (Figure 3A–C). We further computed the pairwise RMSD of systems. Pairwise RMSD analysis helps evaluate whether a simulation has converged to equilibrium by revealing the stabilization of structural deviations over time.

**Figure 4:**
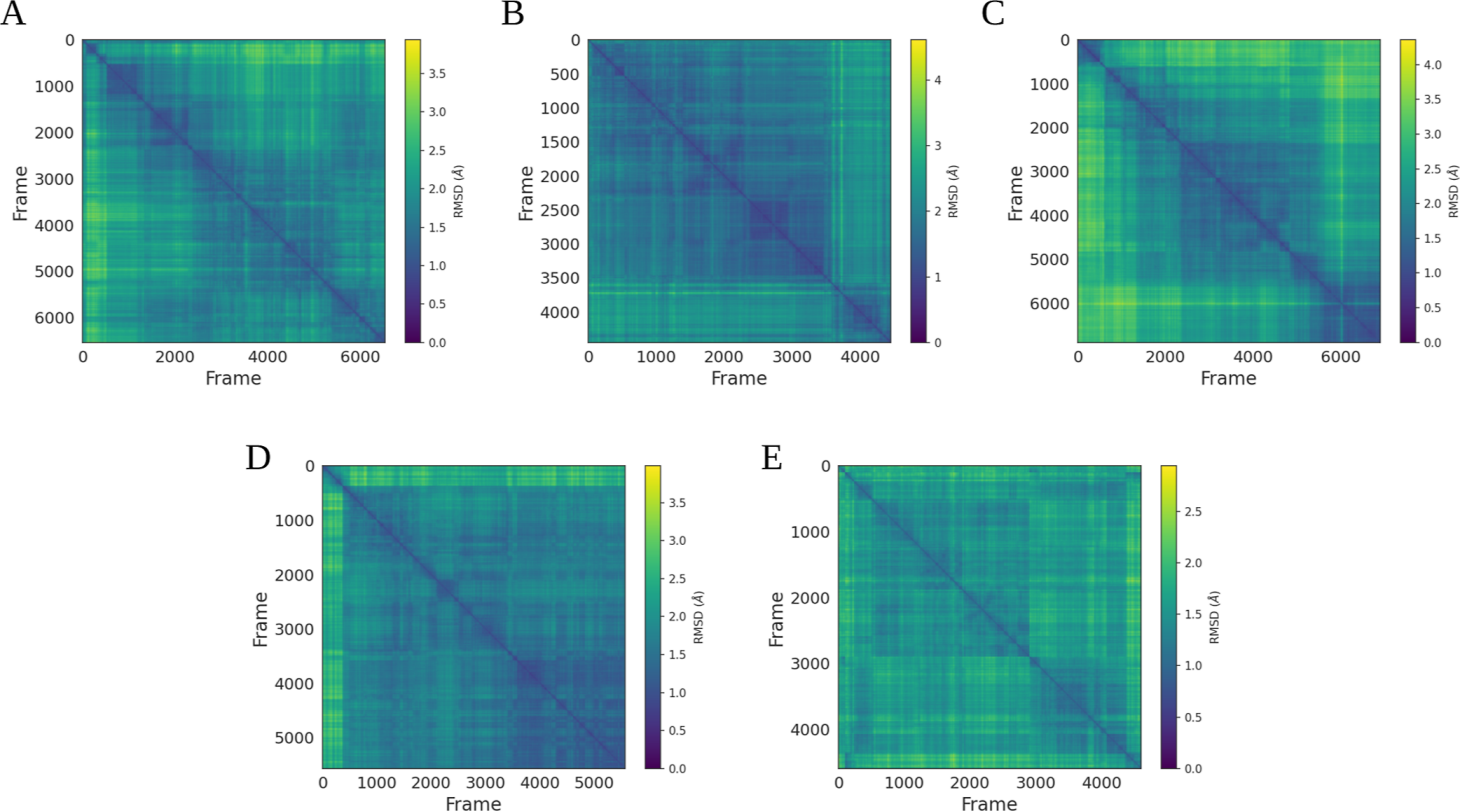
Pairwise RMSD computed for the systems: (A) agonist/3EA, (B) antagonist/EKP, (C) partial agonist/GW0072 (D) agonist/BRL, and (E) agonist/EKP. The number of frames corresponds to 3 μs of simulations.

This analysis of each trajectory against itself (Figure 4) showed low off-diagonal values, particularly in the later stages, indicating that previously explored conformations were revisited, further confirming rapid equilibrium and high stability during the 3 μs of cMD simulations.

To assess structural changes induced by ligand binding, we computed RMSF. Typically, protein secondary structures with flexible regions (such as bends, coils, and turns) become more rigid when stabilized by noncovalent interactions, while flexibility increases when such interactions are fewer. Figure 3D highlights significant fluctuations in residues 263–273, corresponding to the Ω-loop region. RMSF of Ω-loop of antagonist/EKP structure shows a rather small deviation, compared to the full and partial agonist, due to interactions with H12. RMSF values of LBD region remained below 2 Å for all complexes, confirming its stability. Notably, as reported in the literature,^69^ the H12 helix showed increased flexibility in the partial agonist/GW0072 complex due to the lack of direct interaction with H12. We also calculated solvent accessible surface area (SASA) from the MD trajectories. SASA is a crucial metric derived from MD simulations, providing valuable insights into the structural and functional properties of biomolecules. SASA quantifies the surface area of a molecule that is accessible to a solvent, and is a key indicator of molecular hydration, stability, and interaction potential. Figure 3E shows that SASA values for agonist/3EA (ago) and agonist/BRL (coago) were relatively similar, with slightly larger deviations for the latter. The agonist/EKP complex exhibited higher SASA values compared to the antagonist/EKP complex, suggesting a more solvent-exposed state. Together, the RMSF, RMSD, and SASA analyses confirmed the high stability of all protein–ligand complexes, with greater flexibility observed in the H12 helix and Ω-loop region of the partial agonist/GW0072 complex compared to other complexes.

#### 3.1.2 Clustering Analysis

**Figure 5:**
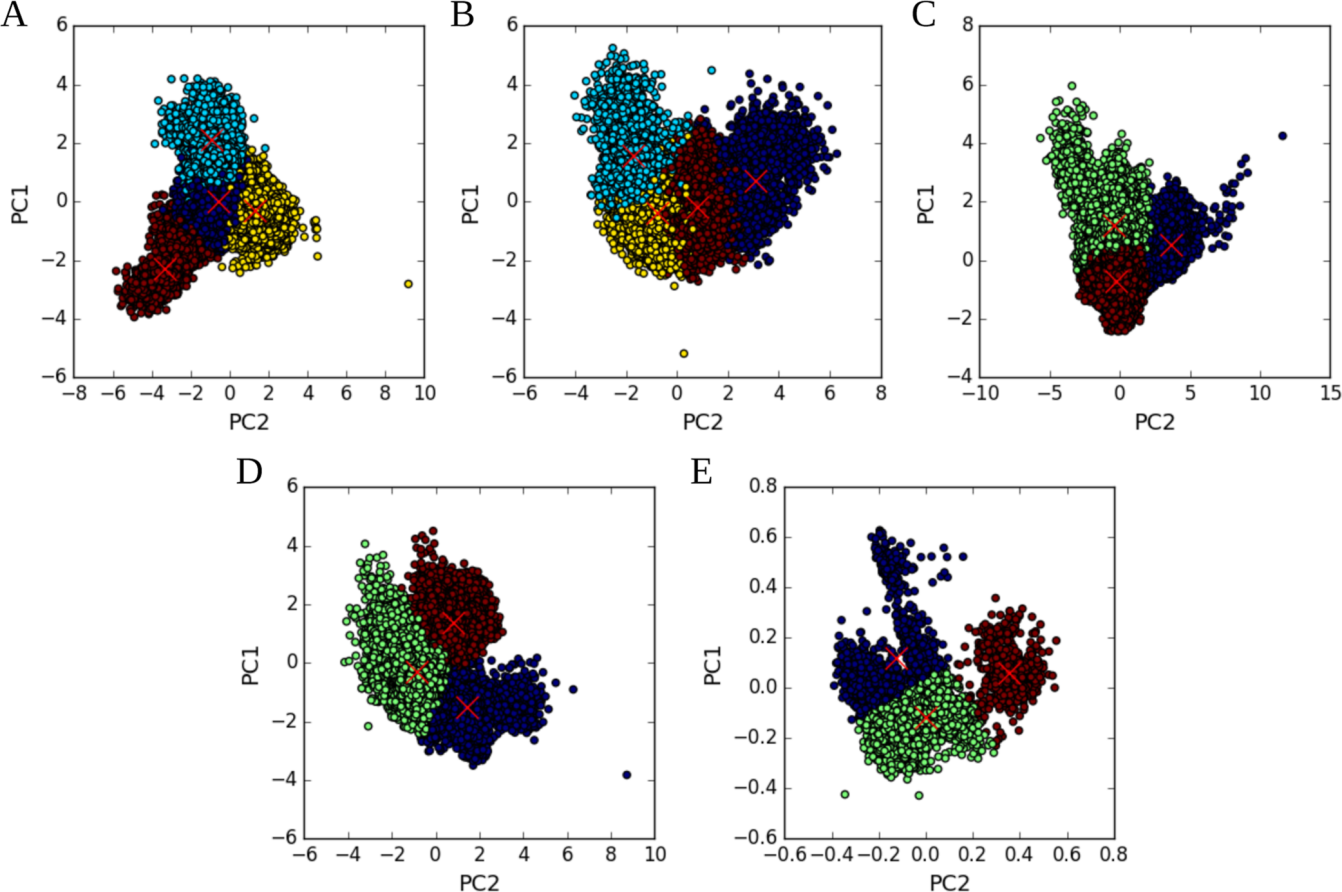
PCA plot showing the first and second principal components, with cluster labels obtained from the K-means++ algorithm applied to the features of: (A) agonist/3EA, (B) antagonist/EKP (C) partial agonist/GW0072 (D) agonist/BRL, and (E) agonist/EKP. Label centroids are highlighted with red crosses. It is worth noting that the 90% of variance is obtained by summing all PCA components, however, the first two represent those components with highest variance.

Following a previously validated protocol,^48^ the dynamic evolution of binding modes throughout the cMD simulations was analyzed using unsupervised machine learning clustering (see Section 2.5), to identify variations in binding arrangements over time, aiming to provide a detailed understanding of these modes. As descriptors, we analyzed several key features for each system, including the evolution of critical interaction distances reported in crystallographic studies, the RMSD of the protein backbone, and the RMSD of interacting residues (see Table S1). These descriptors offer crucial insights into the structural dynamics and stability of the systems under study. By capturing variations in interaction distances and backbone conformations as prior knowledge, these features enable the identification of distinct structural states. Notably, the RMSD of interacting residues provides a detailed view of localized changes of dynamics, enhancing the precision of cluster differentiation.^70^ Data complexity was reduced using principal component analysis (PCA), preserving 90% of the total variance. This process resulted in five principal components for agonist/3EA and partial agonist/GW0072, four for antagonist/EKP and agonist/BRL, and three for the agnoist/EKP system. Figure 5 presents the results of applying ML clustering to the PCA-transformed data, with the number of clusters and their relative weights summarized in Table 1.

**Table 1:** Number of Clusters and Their *W* % Values, i.e. the Percentage Counting the Number of Frames per Cluster

	clusters	*W* %
agonist/3EA	4	48, 30, 21, 1
antagonist/EKP	5	39, 25, 16, 7
partial agonist/GW0072	3	58, 37, 5
agonist/BRL	3	54, 40, 6
agonist/EKP	3	61, 23, 16

#### 3.1.3 Dynamics of Ligand-Bound PPARγ-LBD

**Figure 6:**
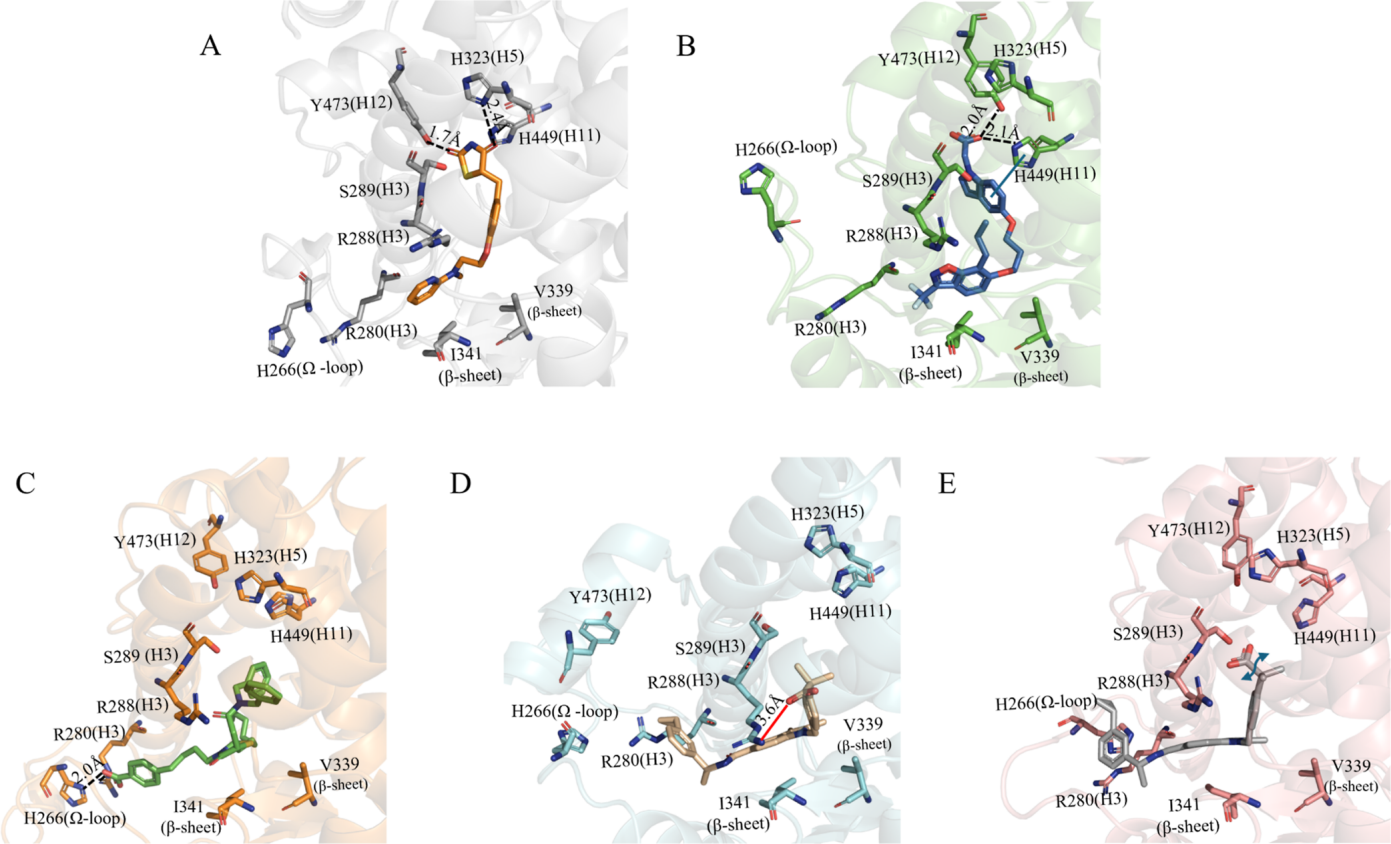
Most representative pose resulting from cluster analysis for (A) agonist/BRL, (B) agonist/3EA, (C) partial agonist/GW0072, (D) antagonist/EKP, (E) agonist/EKP within the PPARγ binding site. Important residues are represented as sticks, while the protein is shown in cartoon representation. H-bonds are illustrated with dashed black lines, and π–π interactions are indicated by a blue line. The blue arrow in panel (E) highlights the mobility of the carboxyl group of EKP. For clarity, hydrogen atoms are omitted.

To investigate the evolution of protein–ligand interactions, we analyzed the clusters generated from our simulations. As noted earlier, RMSD analysis shows the stability of the binding modes throughout the trajectories. This section focuses on the specific interactions between the protein and ligand. Figure 6 presents the binding pose from the most populated cluster, with consistent interactions observed across all clusters. In the PPARγ-LBD complex with the full agonist Rosiglitazone (agonist/BRL—Figure 6A), the thiazole headgroup forms H-bonds with H323 on helix 5 (H5), H449 on helix 11 (H11), and Y473 on helix 12 (H12) of the AF2 domain. Additionally, BRL engages in hydrophobic interactions with I341, V339, R288, and S289 on H3. Similarly, the full agonist 3EA (agonist/3EA—Figure 6B) follows a comparable interaction pattern, forming hydrophobic contacts with I341, V339, R288, and S289 on H3. Its carboxyl group stabilizes H12 through H-bonds with Y473 and engages in π–π interactions with H323 on H5. The binding modes of the antagonist and partial agonists differ from the full agonists, stabilizing the lower part of H3 and remaining distant from H5. The antagonist EKP (Figure 6D) forms a salt bridge with R288 via its carboxyl group, while the rest of the molecule participates in hydrophobic interactions with I341, V339, and S289. The partial agonist GW0072, while maintaining similar hydrophobic contacts, positions its carboxyl group differently, forming H-bonds with R280 on H3 and K266 on the Ω-loop (Figure 6C). Although these interactions are not always seen in X-ray structures of partial agonists, their relevance has been noted for other compounds.^14,71^ In our case study, the antagonist EKP (Figure 6E) adopts a predicted pose similar to the antagonist/EKP complex, establishing hydrophobic interactions with I341, V339, R288, and S289 on H3. However, the rotational flexibility of EKP’s carboxyl group hinders stable interaction with R288, suggesting a lower binding affinity and a reduced ability to stabilize this specific conformation.

#### 3.1.4 Binding Free Energy Calculations for LBD

Binding free energy (Δ*G*_b_) values are typically calculated from representative structures or full MD trajectories.^57,72^ In this study, Δ*G*_b_ values were computed for all complexes based on X-ray structures, except for the agonist/EKP, which was predicted via docking experiments. Specifically, the starting X-ray crystal structures were analyzed only using MM-GBSA, as the ML-ANI approach relies on the statistical determination of the Δ*G*_b_ from more than one configuration. To assess binding dynamics over time, Δ*G*_b_ was also calculated for each centroid conformation extracted from trajectory analysis, with average weight values computed across all centroids. This approach ensures that clusters with higher weights contribute more significantly to the overall analysis. Table 2 demonstrates that Δ*G*_b_^MM-GBSA^ values are consistent across the ligand series.^66^ Prior to simulations, the Δ*G*_b_^MM-GBSA^ scores indicated a favorable binding potential of each small molecule to the PPARγ-LBD. Notably, the Δ*G*_b_^MM-GBSA^ for the antagonist EKP showed minimal variation between the antagonist/EKP and agonist/EKP complexes (−58.83 kcal/mol vs −59.17 kcal/mol). After the simulations, a significant increase in Δ*G*_b_^MM-GBSA^ was observed across all complexes, suggesting substantial conformational adaptation between the ligands and the protein. The most notable finding is the average binding free energy Δ*G*_b_ for the antagonist/EKP complex, which is more favorable than that for the agonist/EKP complex (−97.00 kcal/mol vs −74.82 kcal/mol). This difference is further highlighted by the Δ*G*_b_^ML-ANI^ calculations, which yielded a positive value of 43.18 kcal/mol for the agonist/EKP complex, indicating unfavorable binding. In contrast, consistent with the Δ*G*_b_^MM-GBSA^, the other Δ*G*_b_^ML-ANI^ values are negative, indicating favorable binding.

**Table 2:** Average Binding Energies Computed with MM-GBSA and ML-ANI Calculations for Each Ligand–Receptor System Using Cluster Centroids and the Structures Crystallized via X-ray^a^

	X-ray	centroids	
	Δ*G*_b_^MM-GBSA^	ΔG_b_^MM-GBSA^	ΔG_b_^ML-ANI^
agonist/3EA	–56.75	–66.20	–10.07
antagonist/EKP	–58.83	–97.00	–12.35
partial agonist/GW0072	–104.32	–108.97	–15.96
coactivator-ago/BRL	–52.36	–63.39	–9.76
coactivator-ago/EKP	–59.17	–74.82	43.18

a Units are in kcal/mol.

This positive shift Δ*G*_b_^ML-ANI^ for the agonist/EKP complex may reflect the sensitivity of the ML-ANI approach to changes in the carboxylic group of EKP within the binding pocket.^66^ Overall, these results underscore EKP’s ability to preferentially stabilize the antagonist/EKP conformation over the agonist/EKP conformation, demonstrating the efficacy of the computational workflow.

### 3.2 Exploring PPARγ Ligand Binding Domain Conformational Dynamics

This section examines the conformations of the protein in the presence and absence of modulators to identify potential pathways affecting the surfaces of helices H12, H3, H4, and H5, which comprise the AF-2 domain. To better understand the complex transition of the H12 helix between agonist and antagonist conformations, we investigated three apo conformations derived from removing ligands from the X-ray complexes. Previous NMR studies^69^ have shown that PPARγ-LBD displays regions of flexibility on the microsecond time scale. Therefore, we conducted 3 μs of classical molecular dynamics (cMD) simulations for both apo agonist and apo antagonist systems. Additionally, we applied advanced simulation techniques, WT-MetaD (see Section 2.4) and GaMD (see Section 2.2), to modify the energy potential of selected systems and explore a broader conformational space, as detailed in the Methods Section.

#### 3.2.1 Conformation Dynamics Analysis of PPARγ-LBD

**Figure 7:**
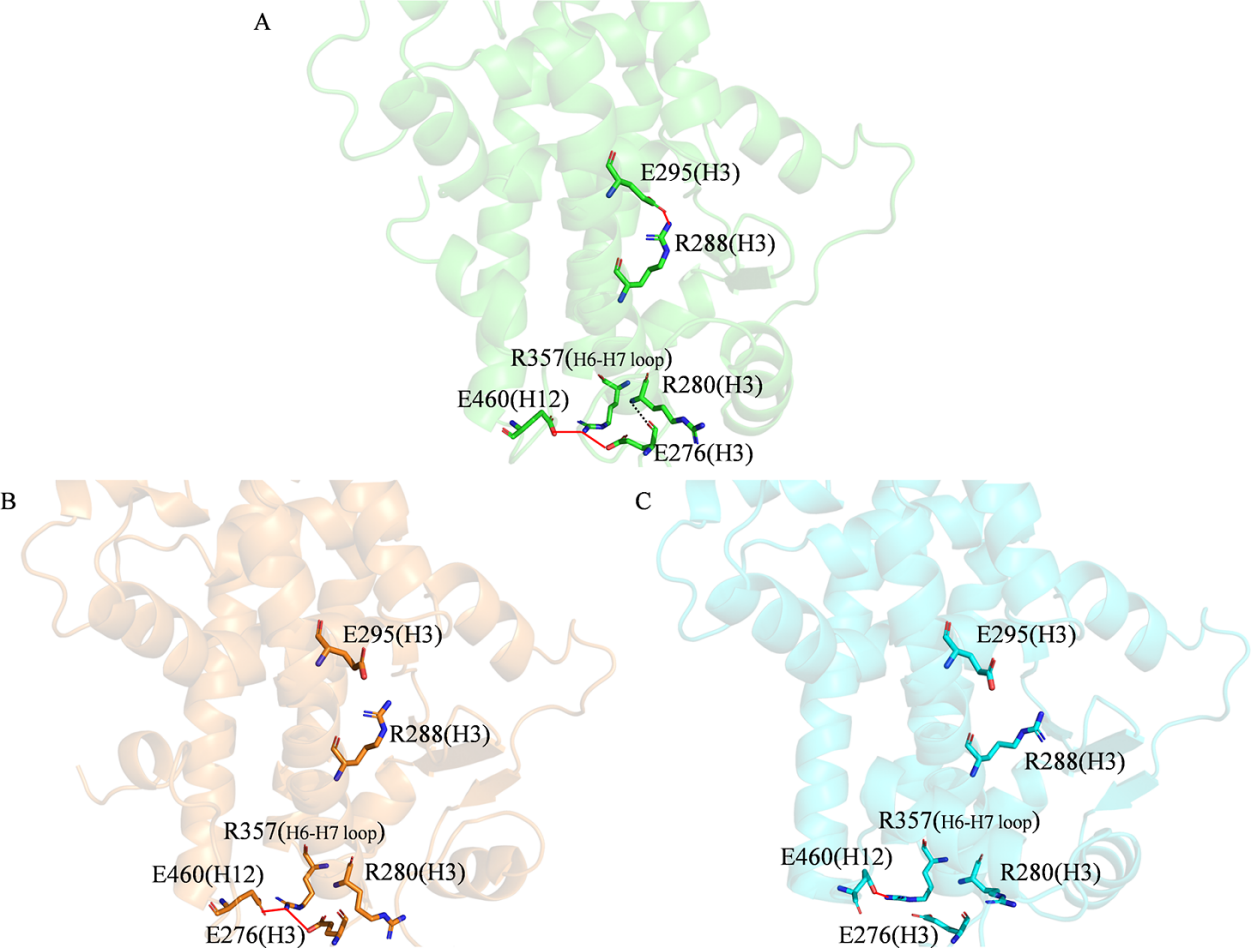
Representative intramolecular H-bond networks for (A) agonist/3EA, (B) partial agonist/GW0072, and (C) antagonist/EKP within PPARγ. Important residues are depicted as sticks, while the protein is depicted in cartoon representation. H-bonds are illustrated with dashed black lines, and salt bridges are indicated by red lines. For clarity, hydrogen atoms are omitted.

**Figure 8:**
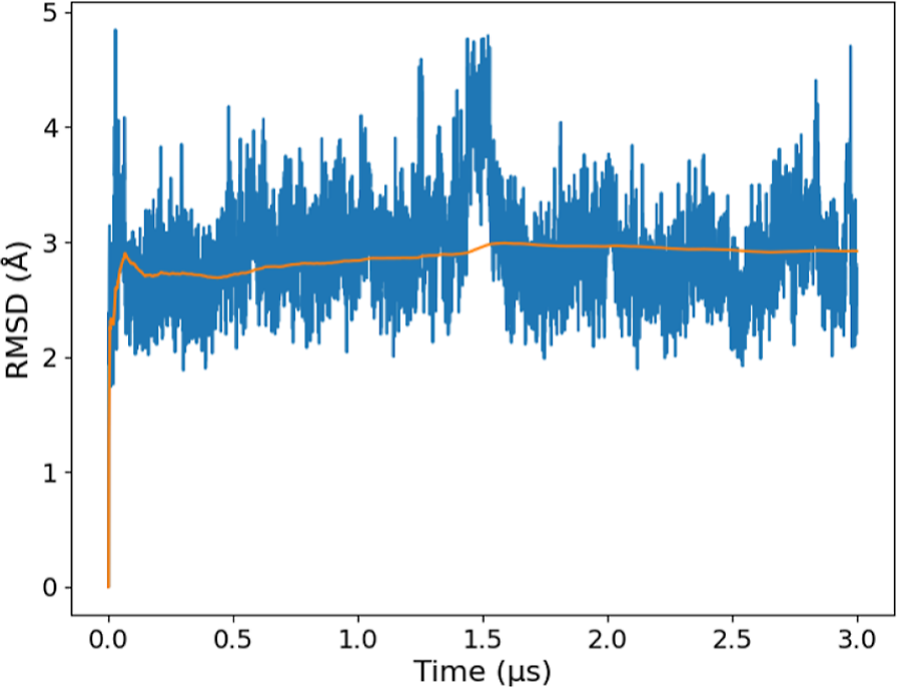
RMSD of helix H12 (backbone) from GaMD (3 μs) simulation of the apo LBD agonist conformation and its cumulative average (orange line).

Surprisingly, during the 3 μs simulations, the apo conformations (apo agonist, apo antagonist, and apo partial agonist) remained globally stable, as evidenced by the pairwise RMSD and RMSF analyses shown in Figures S1 and S2. The RMSF analysis indicated greater flexibility in the N-terminal region (residues 207–209), the Ω-loop, and H12 (RMSF ≥ 3 Å). Despite the overall stability of PPARγ-LBD, we examined the hydrogen-bond network characterizing both complexed and apo conformations to assess the impact of ligand presence. Mutations or binding events can induce long-range effects throughout the protein structure,^73,74^ making the analysis of H-bonds essential for understanding the final conformation and function of the protein. We considered only interactions with an occupancy (the percentage of frames in which each hydrogen bond is present) above 60% as stable and significant. Notably, 14 interhelix H-bonds were consistently observed across all simulated complexes (Table S2). Beyond these core interactions, Table 3 summarizes the key differences in H-bonds across the various conformations and Figure 7 highlights system-specific H-bonds. In the agonist conformation (agonist/3EA) (Figure 7A), interactions with occupancies greater than 70% (Table 3) stabilize H3 (residues 276–303). These interactions include a salt bridge between the side chains of R288 and E295 and an H-bond between the main chains of R280 and E276, which also forms a salt bridge with the side chain of R357, located in the loop between helices 6 (H6) and 7 (H7). R357 additionally forms a salt bridge with the side chain of E460 from H12. Table 3 highlights the differences in interaction patterns between the agonist/3EA conformation and the other complexes. In the partial agonist conformation (partial agonist/GW0072), the interaction between R288 and E295 within H3 is lost. However, it retains the bidentate salt bridge formed by R357 with both E276 and E460, although this occurs less frequently (a 10% difference in hydrogen bond occupancy). The H-bond between the main chains of R280 and E276 persists (Figure 7B). The antagonist conformation (antagonist/EKP) retains only the salt bridge between R357 and E460 (Figure 7C). Notably, the apo conformations exhibit distinct behaviors during the 3 μs simulation. The antagonist conformation regains interactions between R288-E295 and R357-E276, both showing significant occupancy (≥60%), underscoring their importance. This finding aligns with previous experimental data showing that a tripartite salt-bridge network plays a key role in the conformational stability of the receptors.^69^ Furthermore, the orientation of H12 undergoes a slight shift in the apo agonist and apo antagonist conformations. Figure S3 shows a representative frame extracted from MD simulations, illustrating this change, that is critical for H12 due to the absence of the ligand’s stabilizing effect. In the apo agonist conformation (Figure S3A), the side chain of Q454 (H11) interacts with the main chain of L476 (H-bond occupancy = 62.99%) and Y473 (H-bond occupancy = 58.34%). Additionally, Y477 from the main chain interacts with the side chain of Y320 (H5), with an occupancy of 56.61%. In the apo antagonist conformation (Figure S3B), the ligand’s absence leads to a “trap” conformation of H12, similar to that seen in the starting X-ray structure. This state evolves into one where H12 occupies the orthosteric ligand-binding pocket, with Y477 interacting with the side chain of R288 (H3, H-bond occupancy = 63.87%) and L476 with S342 (beta-sheet, H-bond occupancy = 63.87%). These new interactions highlight the critical roles of certain H12 residues in engaging different regions of the ligand-binding domain (LBD) to prevent a high-disorder state in the absence of a ligand during the MD simulations. To gain deeper insights into the motions of H12 in PPARγ-LBD, we extended the classical MD simulations of the apo agonist conformation using Gaussian accelerated molecular dynamics (GaMD). This robust technique captures events occurring over longer time scales that classical MD cannot reach. GaMD enables more extensive conformational sampling without constraints by lowering energetic barriers, thus increasing the likelihood of escaping energetic basins and ensuring accurate calculations of free energy landscapes for biomolecules.^42,75^ Focusing on H12 (residues 466–476), the RMSD profile shown in Figure 8 indicates that H12 remains stable during the 3 μs, despite the bias applied to the entire system.

**Table 3:** Percentage of Hydrogen Bonds Retained throughout MD Simulations^a^

residues pair	ago	par-ago	antago	apo-ago	apo-antago
R357-E460	76.72%	56.29%	88.84%	20.96%	81.18%
R357-E276	73.46%	66.82%	NOB	10.95%	61.18%
R288-E295	88.33%	NOB	NOB	94.91%	78.64%
R280-E276	71.41%	70.74%	NOB	55.53%	48.61%

a NOB is equivalent to not observed. Abbreviations: ago = agonist/3EA; par-ago = partial agonist/GW0072; antago = antagonist/EKP; apo-agonist = apoago; apo antagonist = apo-antago.

#### 3.2.2 H12 Conformations from Metadynamics

**Figure 9:**
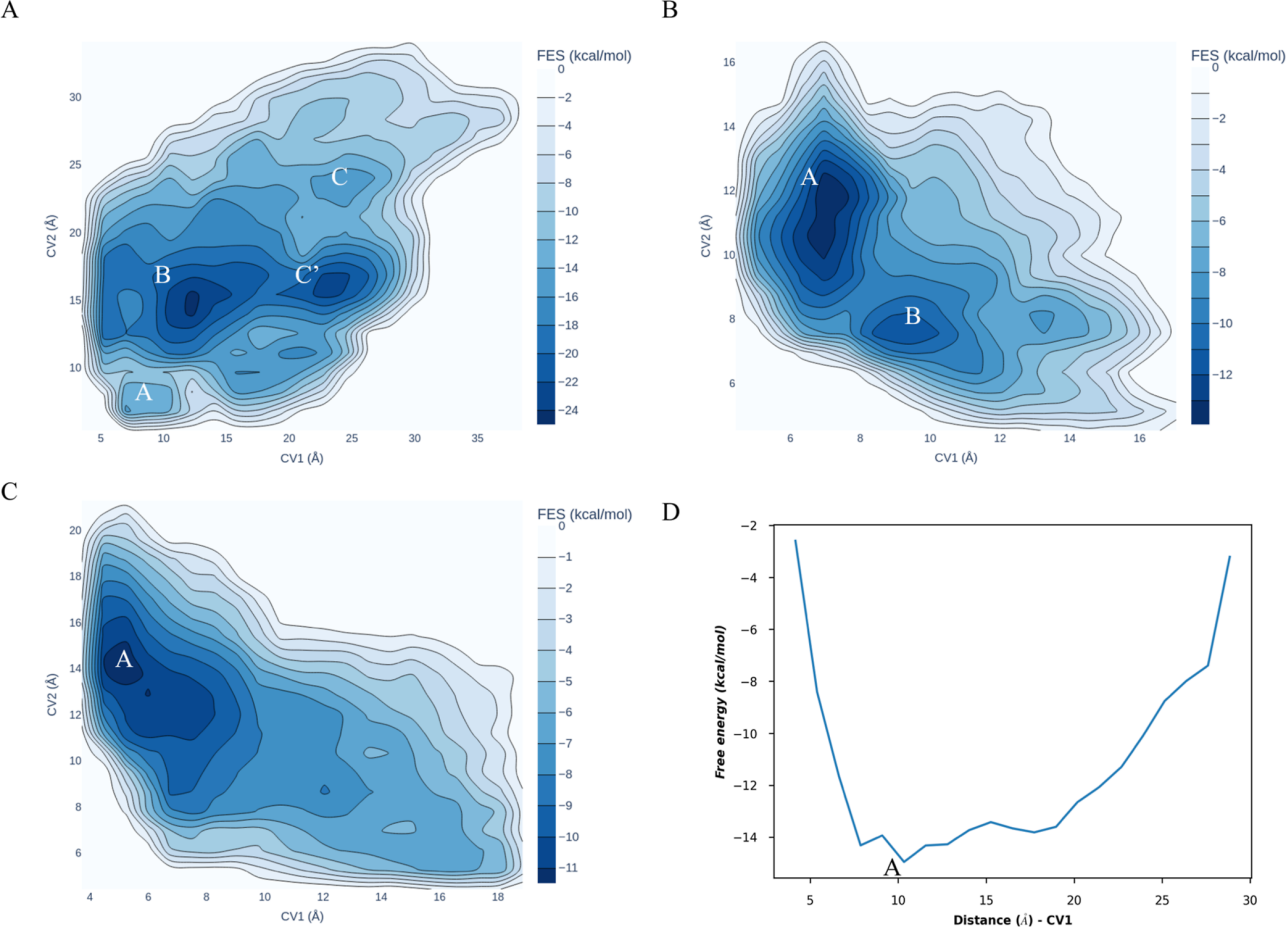
FES plotted against CV1 and CV2 for (A) apo agonist WT-MetaD, (B) agonist/3EA WT-MetaD, and (C) partial agonist/GW0072 WT-MetaD simulations. (D) FES plotted against CV1 for the antagonist/EKP WT-MetaD simulation. The letters A, B, and C denote the relative minima identified in each simulation.

**Figure 10:**
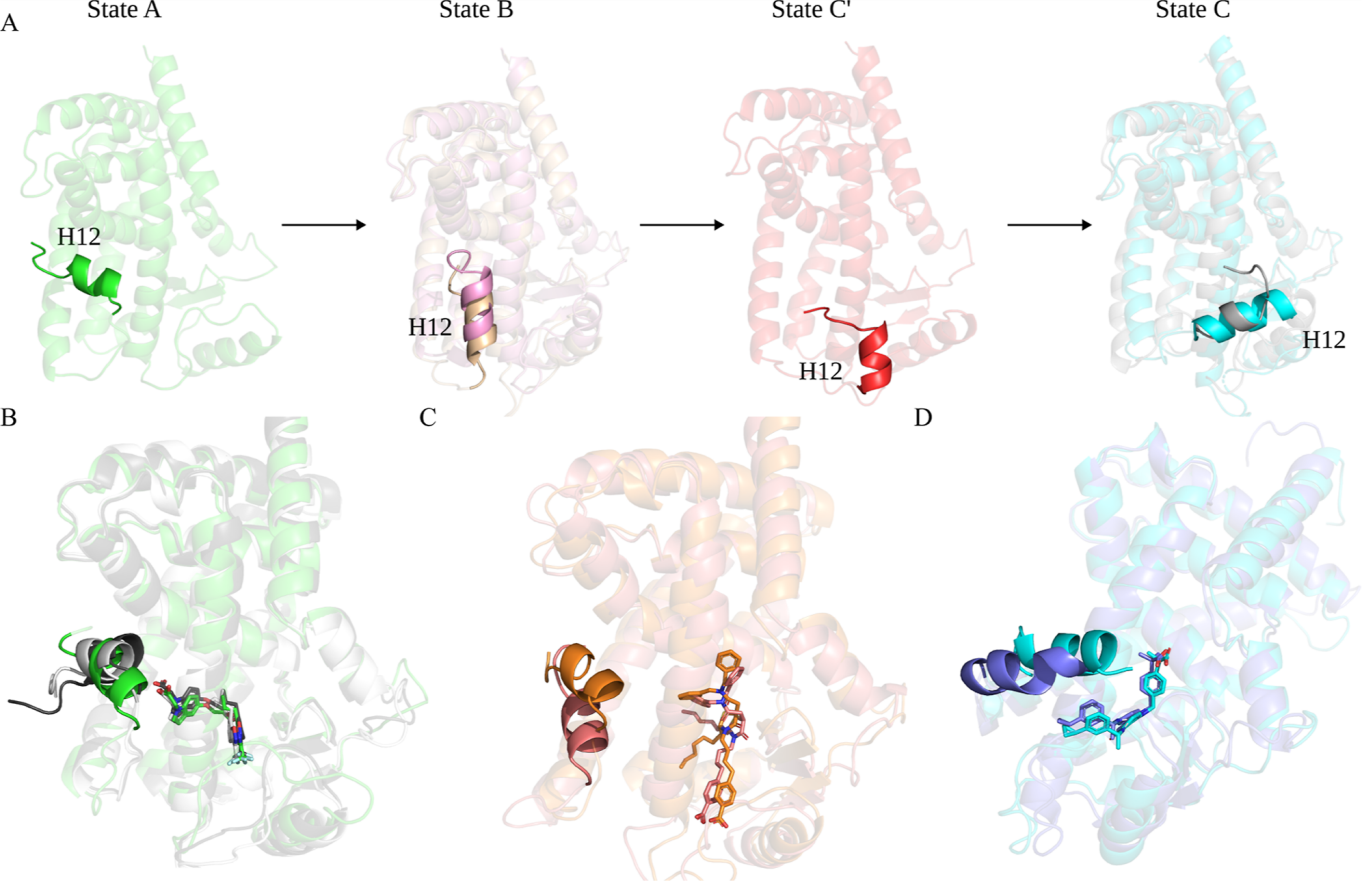
(A) Minima states A, B, C, and C′ observed during apo agonist WT-MetaD simulations, as described in the text. State B (pink) and state C (gray) are superimposed with the crystal structures 1PRG (sand) and 6C5T (light cyan), respectively. (B) Superposition of minima states observed during WT-MetaD simulations of the agonist/3EA complex (initial state: green; minima state A: gray; minima state B: white). (C) Superposition of minima states from the partial agonist/GW0072 WT-MetaD simulation (initial state: orange; minima state A: pink). (D) Superposition of minima states from the antagonist/EKP WT-MetaD simulation (initial state: cyan; minima state A: blue).

Building on the results from classical MD and GaMD, which confirmed that H12 is highly stabilized even in the apo form due to new interactions, we performed three replicas of WT-MetaD simulations to explore H12 mobility. We specifically examined the conformation of apo PPARγ in the agonist state, starting from crystal structures. To avoid artifacts in flexible loops,^46^ we defined CVs based on the distances between the centers of mass of residues Y473 and Y320 (in H5) and Q454 (in H11), since these interactions were responsible for H12 stabilization in previous classical simulations. The 3D free energy surface (FES) was calculated by averaging the FES from all replicas.^46^ Convergence of WT-MetaD was confirmed as the heights of the hills decreased with the flooding of free energy minima (Figure S7A). From Figure 9A, we identified four local minima corresponding to distinct H12 conformations. The most populated state, B, represents a metastable conformation of H12 toward H4, followed by states A and C, which resemble agonist and antagonist conformations. Additionally, we identified state C′, an extended antagonist configuration frequently sampled. State B was the lowest-energy conformation, while the agonist (state A) and antagonist (state C) conformations of H12 had similar energies (Figure 9A). Although the energy difference between the agonist and antagonist H12 conformations suggests a rapid exchange between these states, conformation B is notably stable, implying that full transactivation of the helix occurs through intermediate steps along the pathway A → B → C′ → C. Interestingly, the H12 conformations in states B and C align closely with the H12 structure in the inactive chain B of PDB: 1PRG(15) and the antagonist conformation in PDB: 6C5T(26) (Figure 10A). To gain further insight into the stability of H12 conformations and the role of bound ligands, WT-MetaD simulations were carried out on agonist/3EA, partial agonist/GW0072, and antagonist/EKP. The same protocol as in the simulations of the apo conformation was applied until convergence was achieved (Figure S7B–D). For antagonist/EKP, only one CV was considered, specifically the distance between the centers of mass of residues Y477 (H12) and R288 (H3), since this interaction is responsible for the “trap” conformation in the antagonist state. Inspection of the free energy surfaces (FES) revealed two minima for agonist/3EA (Figure 9B) and one for both partial agonist/GW0072 and antagonist/EKP (Figure 9C,D). Analysis of the conformations relative to these minima indicates that agonist/3EA exhibits H12 conformations similar (ΔRMSD < 1 Å) to the starting conformation (Figure 10B), with the H-bond between the carboxyl group of 3EA and Y473 preserved, confirming the ligand’s role in stabilizing H12. In contrast, the H12 conformation in both partial agonist/GW0072 (Figure 10C) and antagonist/EKP (Figure 10D) shows a shift (ΔRMSD = 2.4 Å) from the initial state. This shift suggests greater flexibility due to the absence of direct interaction between H12 and GW0072/EKP; however, the conformations remain consistent with their respective states (active for the partial agonist and inactive for the antagonist).

## 4 Conclusion

In this study, we explored the complex mechanisms underlying PPARγ-LBD dynamics by integrating all-atom simulations, docking techniques, and rescoring with ML and MM-GBSA. Our primary focus was on X-ray-resolved structures of PPARγ in complex with two agonists (BRL, 3EA), a partial agonist (GW0072), and an antagonist (EKP). To maximize performance and thus reduce computational cost, we combined cMD simulations with the unsupervised ML strategy, following best practices to capture the modulator’s binding mode evolution without loss of significant information.^51,52^

The simulations revealed that PPARγ-LBD undergoes rearrangements that stabilize its conformation with each ligand, as reflected in the significant gain in ΔG_b_^MM-GBSA^ values, exceeding 10 kcal/mol for all complexes except the partial agonist, which gains 5 kcal/mol. Our docking simulations of the EKP antagonist for our case study produced a remarkable result. Although EKP bound stably in the agonist conformation, it failed to interact with the R288 residue, a critical interaction for PPARγ-LBD binding as indicated by previous analyses. This observation supports the efficacy of our computational workflow in distinguishing agonist and antagonist interactions in this specific case. Furthermore, it confirms the EKP inability to induce a rapid conformational shift. This inability was corroborated by the analysis of the conformational dynamics of the PPARγ-LBD complex, suggesting the resilience of the specific conformation induced by the different modulators. Specifically, in addition to 14 interactions shared by all complexes, which are crucial for the inherent stability of PPARγ-LBD, six residues constitute an essential network of H-bonds (R288-E295, R357-E460, R357-E276, and R280-E276) linking H3 and H12. The stability of this network changes depending on whether agonists, partial agonists, or antagonists are present within PPARγ-LBD. A recent study confirms the fundamental role of R357 (located in the H6–H7 loop), which connects H3 and H12 via a tripartite salt bridge with E460 (H12) and E276 (H3).^69^ This study also showed that the E276L mutation, the terminal amino acid of H3 that marks the beginning of the Ω-loop, impacts Ω-loop flexibility by perturbing the tripartite salt bridge. Accordingly, our RMSF results show that the Ω-loop is more flexible in the partial agonist complex (partial agonist/GW0072) than in the agonist (agonist/3EA) and antagonist (antagonist/EKP) complexes, probably due to the differing stability of this tripartite salt bridge. The implication of this different flexibility is not immediately apparent from our MD simulations, but it is known to impact subsequent modulator recruitment.^69^ Previously reported mutagenesis studies support our results, showing that mutations such as R288H and R357A impair the protein’s ability to bind natural ligands and activate transcription.^76,77^ The increased stability of the H-bond network in the agonist conformation (agonist/3EA), compared to the partial agonist (partial agonist/GW0072), correlates with the experimentally observed increased thermodynamically accessible H12 conformations in the partial agonist form. The antagonist EKP induces a distinct conformational state within PPARγ-LBD, characterized by only the R357-E460 interaction while disrupting the crucial tripartite salt bridge (R357-E460-E276) present in the agonist/3EA and partial agonist/GW0072 conformations. This finding could explain the preferential recruitment of corepressors over coactivators, consistent with previous studies. The significance of EKP in disrupting this H-bond network arises from the observation that in the absence of the ligand (apo antagonist conformation), the six key residues (R288, E295, R357, E460, E276, and R280) engage in an H-bond network equivalent to that observed in the apo agonist conformation.

Interestingly, the specific stability of H12 conformations induced by different modulators is confirmed even after ligand removal, as demonstrated in cMD and GaMD simulations. Indeed, H12 retains stability due to interactions with other regions of PPARγ-LBD: H3 in the antagonist conformation and H5/H11 in the agonist conformation. In the apo antagonist conformation, H12 moves into the orthosteric pocket, adopting a repressive state, consistent with findings from paramagnetic relaxation enhancement (PRE) NMR and cross-linking mass spectrometry.^16^

These results support Kojetin et al.’s theory of a mixed-induced fit and conformational selection mechanism.^21^ Our findings suggest that ligands-whether partial, full agonists, or antagonists-bind to pre-existing metastable conformations of PPARγ-LBD, triggering ligand-induced rearrangements that optimize the overall Δ*G*_b_ of the complex. This process leads to form different H-bond network involving key residues (R288, E295, R357, E460, E276, and R280), despite the presence of 14 shared H-bonds across all systems. Although these induced conformations are not directly observable in our MD simulations, evidence from other studies suggests they play a critical role in modulator recruitment. WT-MetaD simulations further support this hypothesis, revealing that complete transactivation of H12 in the apo-agonist form occurs through intermediate steps, progressing via metastable states consistent with experimentally determined structures.^21,69^ Notably, this conformational shift of H12 is not appreciated in ligand-bound structures due to the significant stabilizing effects of modulators. Although the exact mechanism by which different modulators induce the full H12 shift remains elusive, this workflow has proven to be a powerful approach for unraveling PPARγ-LBD conformational dynamics. In particular, this integrated strategy provides a robust framework for studying how full agonists, partial agonists, and antagonists influence PPARγ conformations.

## Data Availability

All of the data underlying this study are available in the manuscript, Supporting Information file, and https://github.com/ emanuelefalbo/AI4PPARg webpage. The MD simulation data were obtained by using the AMBER software suite (AMBER 18) and the structures were visualized by VMD and PyMol. The MD parameter files, force field files, and representative structure files (PDB format) of the major representative complexes (cluster centroids) are available at https://github.com/ emanuelefalbo/AI4PPARg. MM-GBSA, docking, and metadynamics simulations were done with Schrodinger suite (2021), which is a commercially available at https://www.schrodinger.com. ML-ANI software is available at https://github.com/otayfuroglu/deepQM. The in-house protocol is available publicly at https://github.com/ emanuelefalbo/AI4PPARg as form of jupyter notebook. The MDanalysis program can be installed from https://www.mdanalysis.org/. AMBER software suite can be downloaded from https://ambermd.org/ GetAmber.php, VMD from https://www.ks.uiuc.edu/ Research/vmd/and PyMol from https://www.pymol.org/.

## Supporting Information Available

Figures, MD simulation, clustering and analysis details (PDF)
