# Supplementary material for: From Apo to Ligand-Bound: Unraveling PPARγ-LBD Conformational Shifts via Advanced Molecular Dynamics
Source: ACS Omega. 2025 Feb 17;10(13):13303–18. doi: 10.1021/acsomega.4c11128 (PMC11983173; doi:10.1021/acsomega.4c11128)
Supplement: Supplementary file 1 — ao4c11128_si_001.pdf [file ao4c11128_si_001.pdf]

Supporting Information: From Apo to Ligand-Bound: Unraveling PPARγ-LBD Conformational Shifts via Advanced Molecular Dynamics

Emanuele Falbo[†], Pietro Delre[†], and Antonio Lavecchia[*]

Dipartimento di Farmacia, "Drug Discovery Laboratory", Università degli Studi di Napoli "Federico II", via D. Montesano 49, 80131, Napoli, Italy

E-mail: antonio.lavecchia@unina.it

1 MD simulations

Table S1: Features constituting the feature matrix employed for each system during the clustering analysis. The abbreviations *Lig* and *bb* stand for the ligand in the LBD-PPARγ and backbone atoms of protein, respectively, while COM refers to the center of mass. The names of the atoms involved in the bonding interactions are followed by the @ symbol.

System	Distances	RMSD
agonist/3EA	Y473@HH-Lig@030, H323-Lig@O31	bb PPARγ atoms, Lig
	H449$_{COM}$-Lig$_{COM/pyrrole}$, H449$_{COM}$-Lig$_{COM/benzene}$	Y473, H323
partial agonist/GW0072	S342@H-Lig@O1A, R280@HE-Lig@03M	bb PPARγ atoms, Lig
	F264$_{COM}$-Lig$_{COM}$	F264, S342
antagonist/EKP	R288-Lig@O1A, G283-S464	bb PPARγ atoms, Lig
	F287$_{COM}$-Lig$_{COM}$	R288, F287, G283, S464
agonist/BRL	F287$_{COM}$-Lig$_{COM}$, R288@HH-Lig@N3	bb PPARγ atoms, Lig, F287, R288
agonist/EKP	F287$_{COM}$-Lig$_{COM}$	bb PPARγ atoms, Lig, F287

Table S2: Table showing the inter-helix H-bonds shared by the various conformations during the simulations expressed as percentages. Note that the starting number of sequence is from one from our topology file, while within the crystal structure is from 206. For example, the first line corresponds to the interaction between: VAL239-M and MET248-M. M and S stand respectively for main and side chain.

		3EA	BRL	072	EKP	apo ago	apo pago	apo antag
VAL133-M	MET142-M	74.52%	74.45%	75.07%	60.39%	79.05%	73.78%	53.12%
TYR93-S	ALA179-M	73.05%	75.73%	79.72%	60.64%	75.72%	65.17%	60.87%
THR143-S	ILE43-M	81.46%	74.94%	83.73%	68.96%	82.37%	75.99%	84.15%
MET142-M	VAL133-M	79.47%	81.04%	78.15%	81.45%	73.86%	75.99%	59.12%
HIP219-S	ASP177-S	85.95%	50.1%	88.74%	84.96%	85.46%	85.46%	84.36%
GLN214-S	ASP177-S	95.11%	85.54%	94.13%	95.49%	95.08%	95.08%	94.52%
ASN218-S	ASP177-S	82.72%	63.64%	81.34%	81.94%	84.04%	84.04%	84.19%
ASN218-S	ASP174-S	79.72%	52.5%	69.27%	81.42%	79.98%	79.98%	76.58%
ARG74-S	GLU53-S	83.44%	62.44%	52.5%	32.5%	73.77%	75.33%	80.78%
ARG237-S	GLU118-S	75.43%	84.62%	81.3%	84.45%	79.43%	77.45%	87.22%
ARG191-S	GLU118-S	93.8%	97.83%	97.49%	96.91%	91.86%	98.5%	97.37%
ARG144-S	GLU159-S	86.58%	86.98%	78.13%	84.75%	85.66%	77.01%	38.94%
ARG144-M	ASP131-M	87.76%	89.11%	84.87%	87.27%	82.46%	82.77%	69.94%
ALA25-M	ASP175-S	87.32%	88.09%	67.13%	75.43%	91.38%	80.67%	68.45%

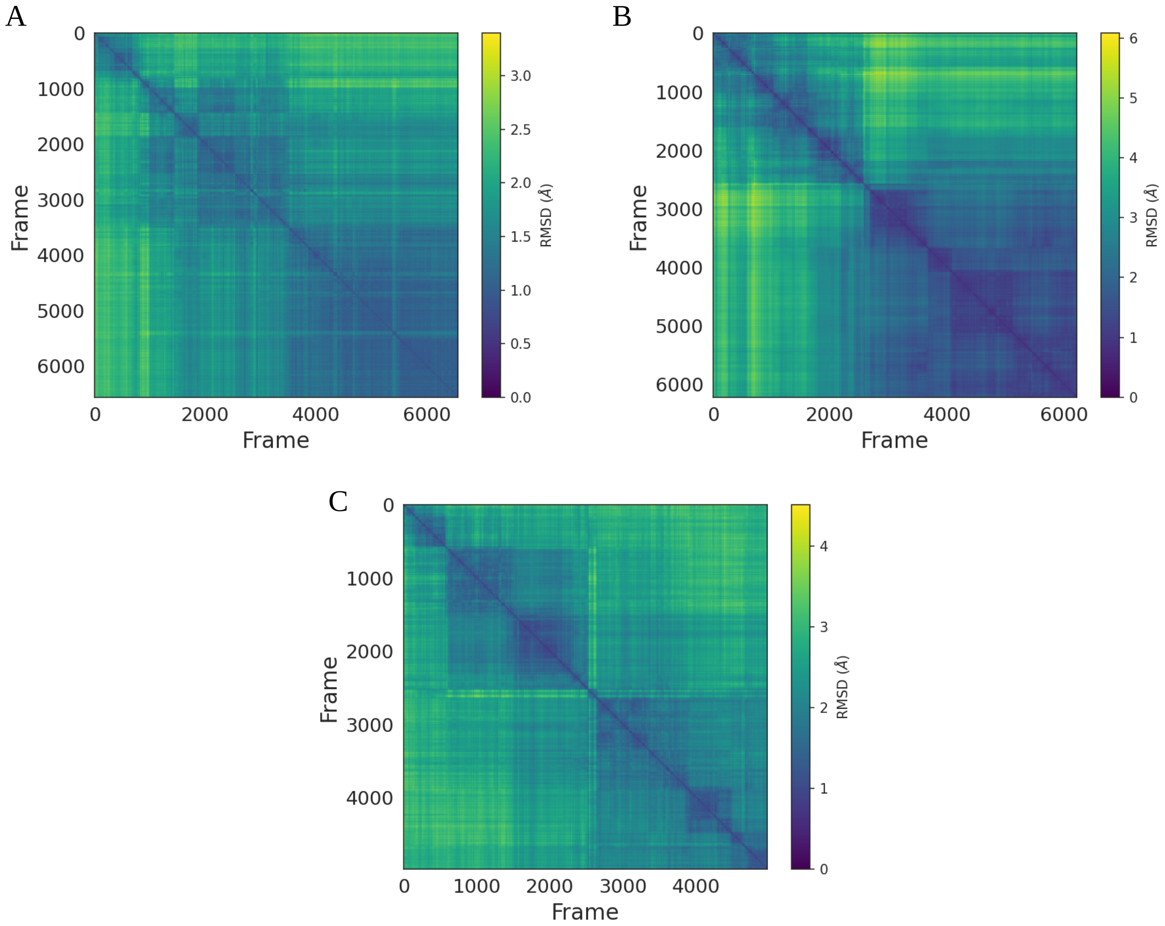

Figure S1: Pairwise RMSD for apo forms of (A) agonist, (B) antagonist, and (C) partial agonist MD simulations of PPARγ-LBD. The number of frames correspond to 3 μs of simulations.

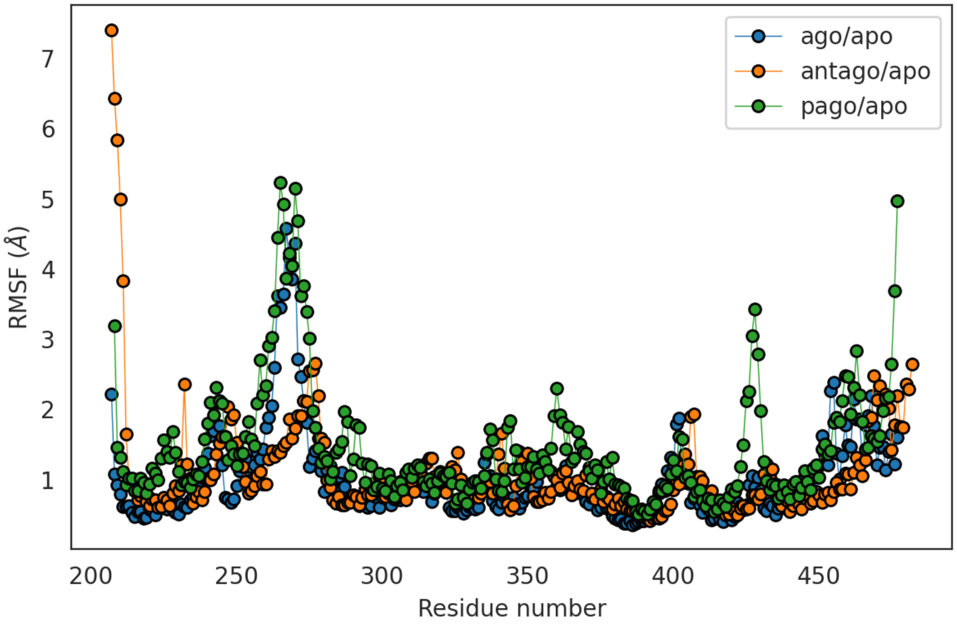

Figure S2: RMSF for apo conformations of agonist (ago/apo), antagonist (antago/apo), and partial agonist (pago/apo).

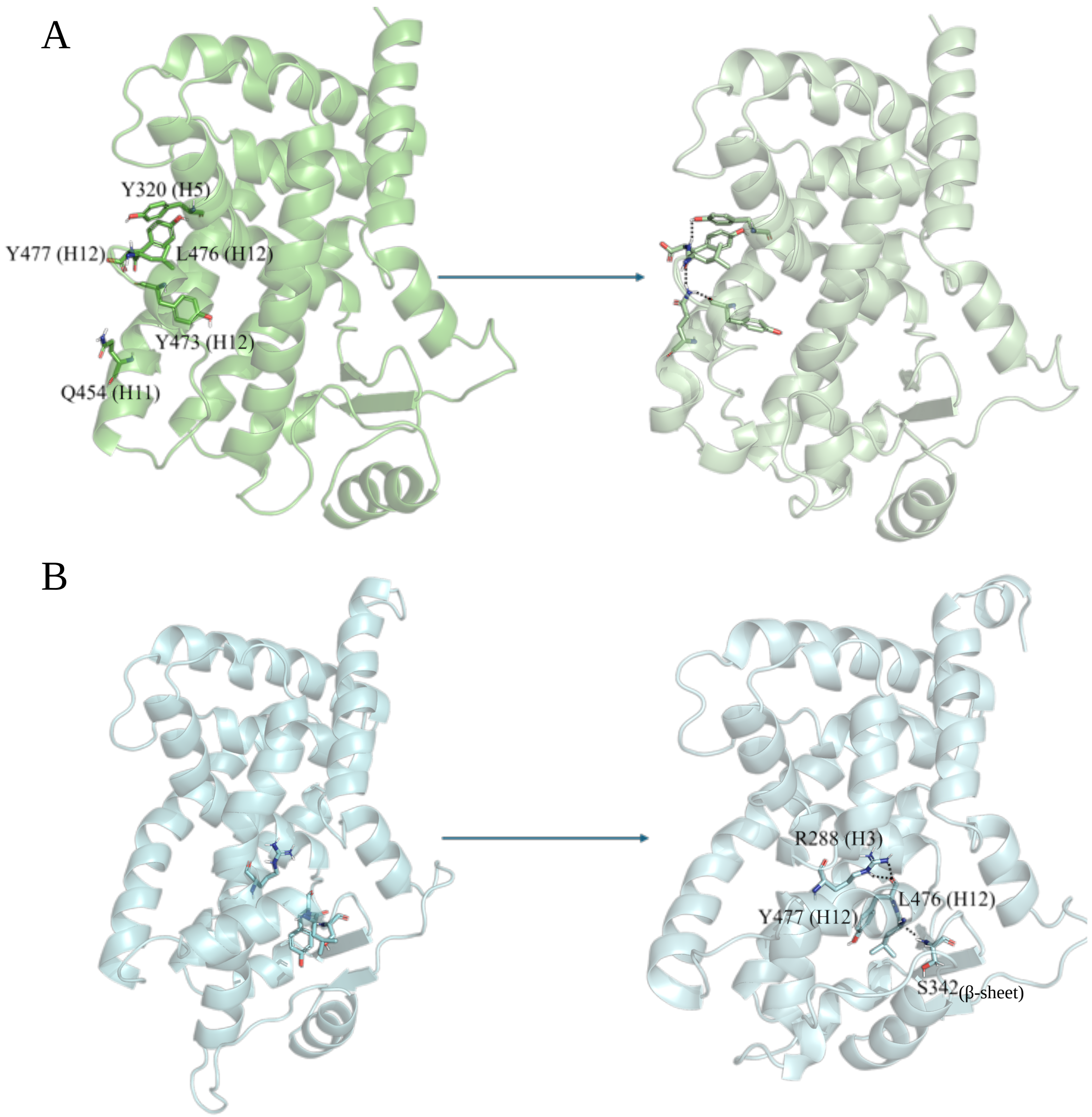

Figure S3: Initial and representative frames showing the H12 conformational chang. (from left to right) of classical MD simulations for apo agonist (**A**) and antagonist (**B**).Important residues are depicted as sticks, while the protein is shown in cartoon representation. H-bonds are illustrated with dashed black lines. For clarity, non polar hydrogen atoms are omitted.

2 Clustering Analysis

SilhouetteScore (SI), DunnIndex (DI), Calinski-Harabasz score (pSF), and Within Sum of Squares error (WSS) are expected to reach their highest values with the parameter set (specifically the value of k) that results in the most effective clustering. Meanwhile, WSS is evaluated for any changes in its slope. Hence, the optimal number of clusters is determined when at least three out of these four metrics meet the desired criteria.

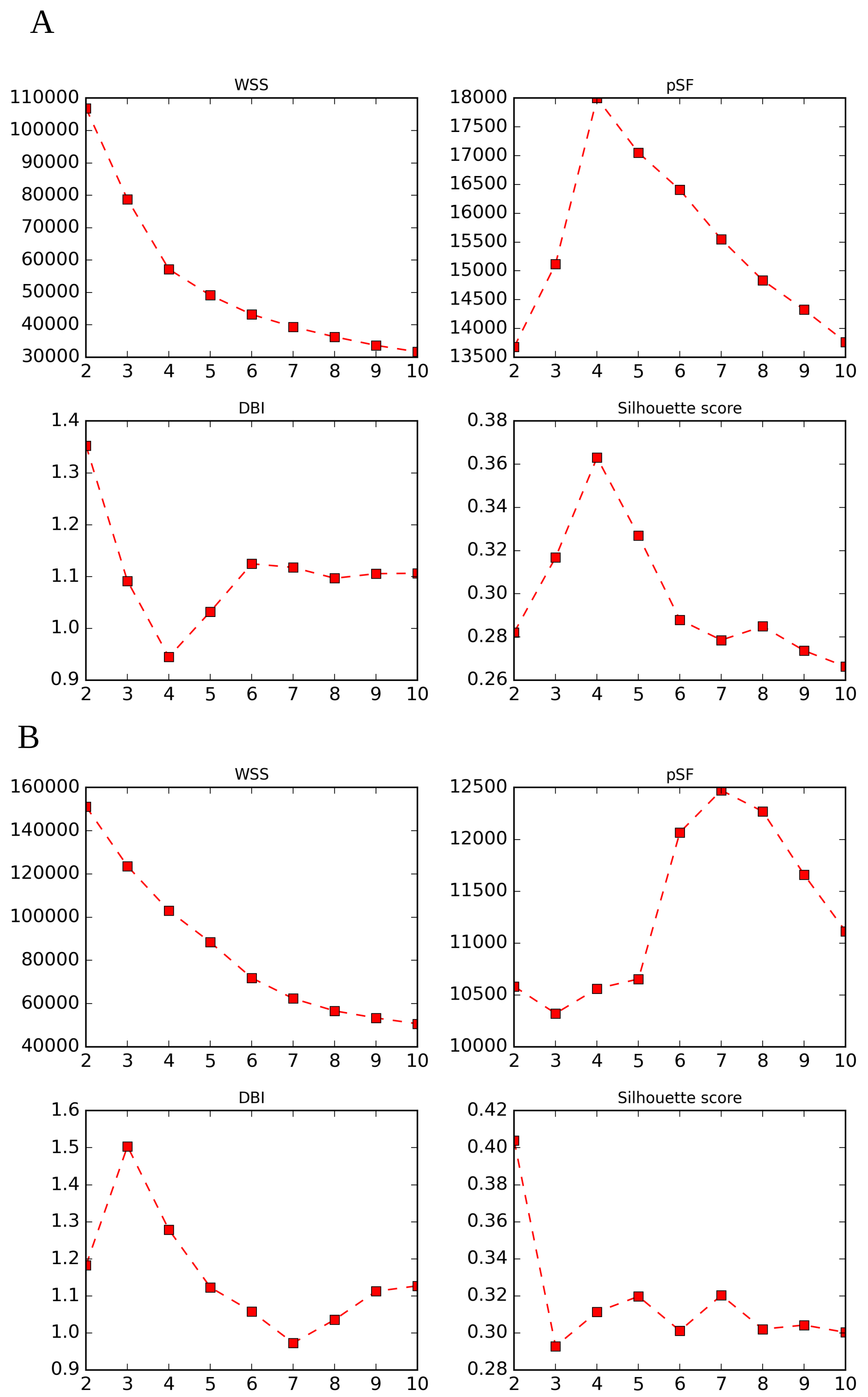

Figure S4: Within cluster sum of squares error (WSS), Calinski- Harabasz score (psf), Dunn Index (DI), and Silhouette coefficient (SI) values (y-axis) computed for increasing k (x-axis) for **(A)** agonist/3EA and **(B)** partial agonist/GW0072 PCA feature spaces.

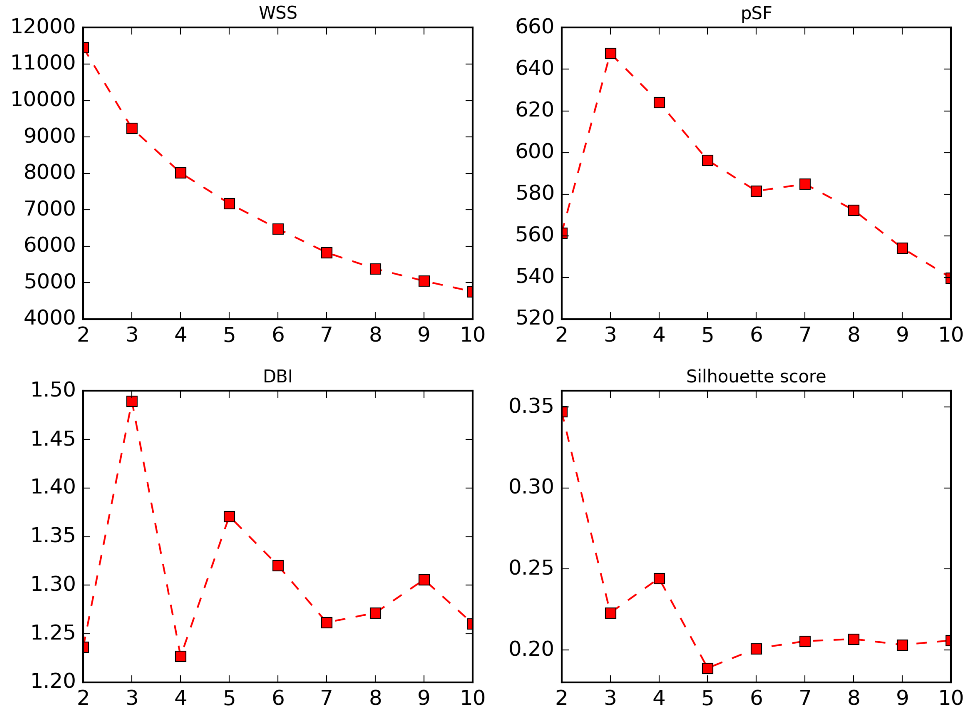

Figure S5: within cluster sum of squares error (WSS), Calinski- Harabasz score (psf), Dunn Index (DI), and Silhouette coefficient (SI) computed for increasing k (x-axis) for antagonist/EKP MD simulations

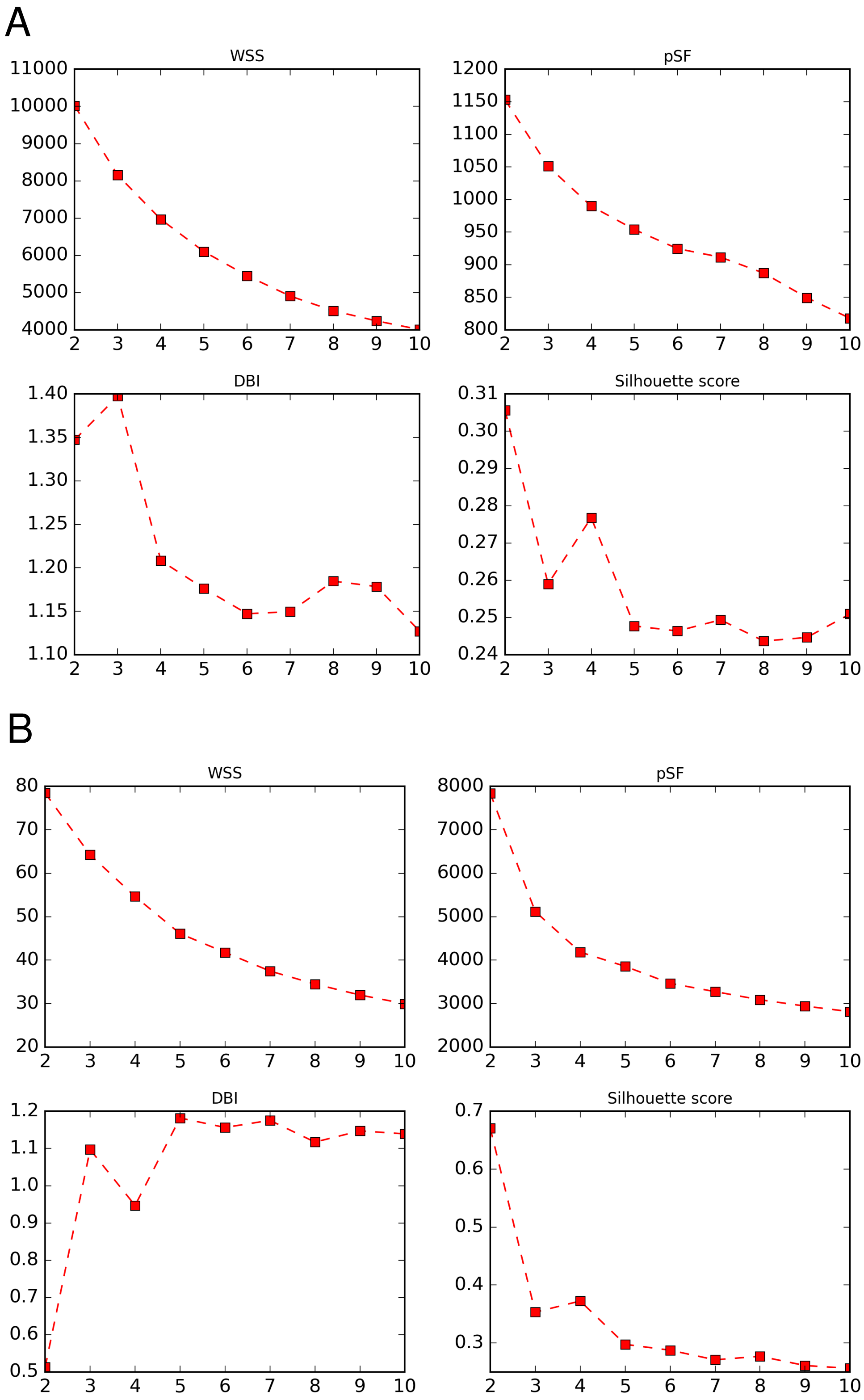

Figure S6: within cluster sum of squares error (WSS), Calinski- Harabasz score (psf), Dunn Index (DI), and Silhouette coefficient (SI) computed for increasing k (x-axis) for **(A)** agonist/BRL and **(B)** agonist/EKP PCA feature spaces.

3 Metadynamics

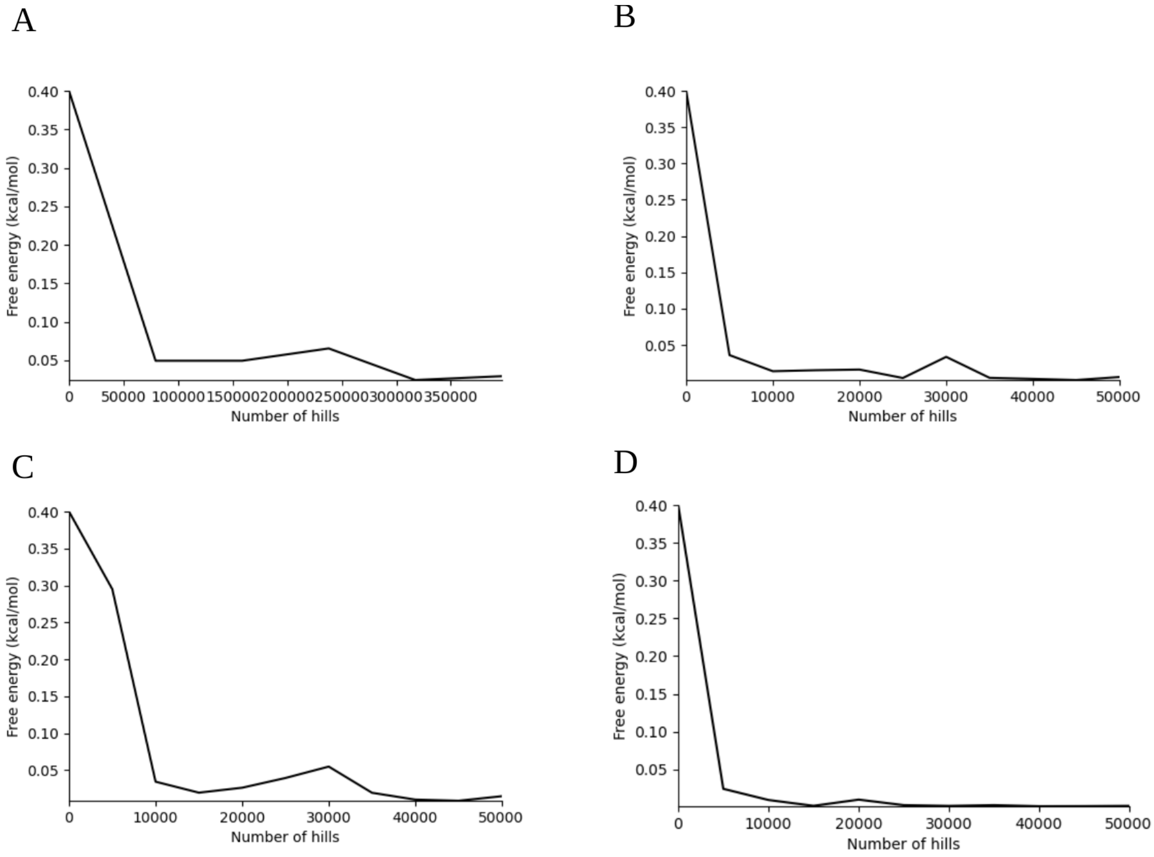

Figure S7: Error heights as function of number of hills for each metadynamics simulation averaged over the three replicas. A) apo agonist, B) agonist/3EA, C) partial agonist/GW0072, and D) antagonist/EKP.
